# The impact of relaxing restrictions on take-home doses during the COVID-19 pandemic on program effectiveness and client experiences in opioid agonist treatment: a mixed methods systematic review

**DOI:** 10.1186/s13011-023-00564-9

**Published:** 2023-09-30

**Authors:** Alison Adams, Sarin Blawatt, Tianna Magel, Scott MacDonald, Julie Lajeunesse, Scott Harrison, David Byres, Martin T. Schechter, Eugenia Oviedo-Joekes

**Affiliations:** 1 Centre for Health Evaluation & Outcome Sciences, Providence Health Care, St. Paul’s Hospital, 575-1081 Burrard St., Vancouver, BC V6Z 1Y6 Canada; 2 School of Population and Public Health, University of British Columbia, 2206 East Mall, Vancouver, BC V6T 1Z3 Canada; 3 Providence Health Care, Providence Crosstown Clinic, 84 West Hastings Street, Vancouver, BCV6B 1G6 Canada; 4 Provincial Health Services Authority, 200-1333 W Broadway, Vancouver, BC V6H 4C1 Canada

**Keywords:** Substance use, Opioid use disorder, Opioid agonist treatment, COVID-19

## Abstract

**Background:**

The COVID-19 pandemic led to an unprecedented relaxation of restrictions on take-home doses in opioid agonist treatment (OAT). We conducted a mixed methods systematic review to explore the impact of these changes on program effectiveness and client experiences in OAT.

**Methods:**

The protocol for this review was registered in PROSPERO (CRD42022352310). From Aug.–Nov. 2022, we searched Medline, Embase, CINAHL, PsycInfo, Web of Science, Cochrane Register of Controlled Trials, and the grey literature. We included studies reporting quantitative measures of retention in treatment, illicit substance use, overdose, client health, quality of life, or treatment satisfaction or using qualitative methods to examine client experiences with take-home doses during the pandemic. We critically appraised studies using the Mixed Methods Appraisal Tool. We synthesized quantitative data using vote-counting by direction of effect and presented the results in harvest plots. Qualitative data were analyzed using thematic synthesis. We used a convergent segregated approach to integrate quantitative and qualitative findings.

**Results:**

Forty studies were included. Most were from North America (23/40) or the United Kingdom (9/40). The quantitative synthesis was limited by potential for confounding, but suggested an association between take-home doses and increased retention in treatment. There was no evidence of an association between take-home doses and illicit substance use or overdose. Qualitative findings indicated that take-home doses reduced clients’ exposure to unregulated substances and stigma and minimized work/treatment conflicts. Though some clients reported challenges with managing their medication, the dominant narrative was one of appreciation, reduced anxiety, and a renewed sense of agency and identity. The integrated analysis suggested reduced treatment burden as an explanation for improved retention and revealed variation in individual relationships between take-home doses and illicit substance use. We identified a critical gap in quantitative measures of patient-important outcomes.

**Conclusion:**

The relaxation of restrictions on take-home doses was associated with improved client experience and retention in OAT. We found no evidence of an association with illicit substance use or overdose, despite the expansion of take-home doses to previously ineligible groups. Including patient-important outcome measures in policy, program development, and treatment planning is essential to ensuring that decisions around take-home doses accurately reflect their value to clients.

**Supplementary Information:**

The online version contains supplementary material available at 10.1186/s13011-023-00564-9.

## Introduction

Opioid use disorder affects an estimated 21.4 million people worldwide [1]. It is associated with significant morbidity and mortality, attributable in part to the stigmatization, social marginalization, and criminalization of people who access the unregulated drug supply [2, 3]. Regionally, opioid use disorder is most prevalent in high-income North America [4]. In 2022, a total of 83,827 deaths in the United States and 7,328 deaths in Canada were attributed to opioid toxicity [5, 6]. This is a substantial increase over 2016, when 43,149 deaths were reported in the United States and 2,831 in Canada [5, 6]. The severity of the overdose crisis in this region of the world is the result of historical overprescribing, social factors, and an unregulated drug supply that is heavily contaminated with fentanyl, benzodiazepines, and other adulterants [7–9].

Opioid agonist treatment (OAT) using methadone or buprenorphine is an effective and well-established approach to reducing the harms associated with opioid use disorder [10–13]. Both methadone and buprenorphine suppress use of unregulated opioids when prescribed at adequate doses [11, 14] and are associated with substantial reductions in rates of fatal and non-fatal overdose [13, 15]. Despite these benefits, retention in OAT is low; it ranges from 19 to 86% at six months, with a median retention rate of 58% [16]. Mortality rates rise steeply after treatment cessation [13].

Burdensome treatment conditions, particularly for clients on methadone, may contribute to low retention in OAT [17]. These conditions commonly include supervised dosing, in which OAT clients must travel to their clinic or pharmacy each day so that their medication can be ingested under the observation of a health care provider [18]. Take-home doses, which can be carried out of the clinic and stored safely elsewhere, may be granted to clients who meet specific criteria.

In the United States, pre-pandemic guidelines for methadone programs required clients to meet eight criteria reflecting ‘stability’ and to remain in treatment for a minimum of six months before becoming eligible to receive more than two take-home doses per week [19]. Factors affecting eligibility for take-home OAT in other jurisdictions include time in treatment, abstinence from illicit substance use, housing stability, distance from the treatment facility, and provider discretion [18, 20].

Restrictions on take-home doses are driven by concerns over the potential for diversion, injection, and overdose [21]. Methadone is approached with particular caution; as a full agonist with a long half-life, it has the potential to cause serious respiratory depression if taken in excess or in conjunction with alcohol, unregulated opioids, or other sedatives [21]. For this reason, careful titration is necessary to initiate methadone safely. However, systematic reviews of supervised versus unsupervised dosing have found insufficient evidence to determine whether restrictions on take-home doses are effective in reducing diversion [22, 23]. Recent research has drawn attention to the role of unmet treatment need in the market for diverted medication [24–26] and highlighted the potential for benefits as well as harms [27, 28].

Though some OAT clients appreciate the structure of daily supervised dosing [29, 30], inflexible restrictions on take-home doses have repeatedly been identified as a source of dissatisfaction with treatment [31]. In addition to “[obstructing] the basic day-to-day functioning of life” [32] (p. S118), supervised dosing has been described as humiliating, degrading, and stigmatizing [29, 33, 34]. Commentators have argued that supervised dosing is part of a treatment paradigm that reinforces institutional stigma and power imbalances, serving as a form of social control as well as a medical intervention [35–38].

The COVID-19 pandemic led to the relaxation of restrictions on take-home doses on an unprecedented scale. The risks of viral infection to clients and providers in medical settings, as well as the dangers of treatment discontinuation for clients who might stop OAT to avoid exposure to COVID-19, were deemed to outweigh the potential harms of take-home doses. Regulations and guidelines to encourage use of take-home doses during the pandemic were developed in Canada [39], the United States [40, 41], Australia [42], England [43], Spain [44], Italy [45], and India [46]. Other changes to OAT during COVID-19 included the suspension of urine testing or a reduction in testing frequency, increased emphasis on naloxone distribution, medication delivery for clients in isolation or quarantine, and the use of virtual care in place of in-person visits [39, 41–43, 45, 46]. Though implementation of the new flexibilities around take-home doses varied [47], their introduction created an unparalleled opportunity to assess the impact of relaxing restrictions on take-home doses in OAT.

Previous reviews of changes to take-home guidance during COVID-19 have focused on providers’ experiences [48] and changes within the United States [49]. To our knowledge, this is the first systematic review of international scope to focus on how relaxing restrictions on take-home doses during the COVID-19 pandemic affected program effectiveness and client experiences in OAT. Results from this study can support clinicians, policymakers, and stakeholders in making informed decisions around the implementation and expansion of take-home doses in OAT.

## Methods

### Design

We conducted a mixed methods systematic review to address the following questions:

- Q1 (quantitative): What was the impact of relaxing restrictions on take-home doses during the COVID-19 pandemic on program effectiveness in OAT, as defined by (1) retention; (2) illicit substance use; (3) fatal and non-fatal overdose; (4) client health (e.g., measures of physical, mental, or emotional health); (5) quality of life; and (6) treatment satisfaction?
- Q2 (qualitative): What was the impact of relaxing restrictions on take-home doses during the COVID-19 pandemic on clients’ experiences with OAT?
- Q3. What are the integrated findings of Q1 and Q2, and what are their implications for OAT?

Mixed methods approaches have the potential to generate a more complete and nuanced understanding of a phenomenon than quantitative or qualitative evidence alone. Qualitative evidence can suggest explanations for quantitative findings, help policymakers predict the impact of an intervention in a specific context, and illuminate aspects of human experience that are not captured by quantitative research [50]. We used a convergent segregated approach in which the quantitative synthesis (Q1) and qualitative synthesis (Q2) are conducted separately before being integrated through ‘configured analysis’ (Q3) [51]. Reporting of the methods was guided by the PRISMA and PRISMA-S statements for reporting systematic reviews and the Synthesis Without Meta-analysis (SWiM) reporting guideline [52–54] (Additional file 1). The protocol for this review was registered in PROSPERO (CRD42022352310; https://www.crd.york.ac.uk/prospero/).

### Search strategy

We used the PICO (Population, Intervention, Comparator, Outcomes) and PICo (Population, phenomenon of Interest, Context) frameworks to structure our search strategy and define our inclusion criteria (Table 1).

**Table 1:** PICO and PICo criteria for review questions Q1 and Q2

Q1: What was the impact of relaxing restrictions on take-home doses during the COVID-19 pandemic on program effectiveness in OAT, as indicated by (1) retention; (2) illicit substance use; (3) fatal and non-fatal overdose; (4) client health; (5) quality of life; and (6) treatment satisfaction?	**P (Population):** People receiving OAT via any route of administration (e.g., oral, sublingual, buccal, injectable)
	**I (Intervention):** Relaxation of restrictions on take-home doses of OAT during the COVID-19 pandemic^a^
	**C (Comparator):** (1) No comparator OR (2) restrictions on take-home doses prior to the COVID-19 pandemic
	**O (Outcomes):** Program effectiveness, as indicated by incidence of (1) retention; (2) illicit substance use; (3) fatal and non-fatal overdose; (4) client health; (5) client quality of life; and (6) client treatment satisfaction
Q2: What was the impact of relaxing restrictions on take-home doses during the COVID-19 pandemic on clients’ experiences with OAT?	**P (population):** People receiving OAT via any route of administration (e.g., oral, sublingual, buccal, injectable)
	**I (phenomenon of Interest):** Client experience (e.g. satisfaction with treatment, relationship with provider, self-efficacy, alignment of service with personal treatment goals, other patient-reported outcomes)
	**Co (Context):** Relaxation of restrictions on take-home doses of OAT during the COVID-19 pandemic^a^

*Abbreviations*: *OAT* opioid agonist treatment

^a^As specified in the review protocol, we included studies in which relaxed restrictions on take-home doses formed part of a broader intervention or context

The search strategy was developed by a member of the research team with expertise in systematic searching (AA) and reviewed by a professional research librarian. Substantive elements of the search strategy for this review were used in a previously published review [48]. We restricted all searches to articles published after January 1, 2020 because the review focuses on actions taken in response to the COVID-19 pandemic.

We searched six electronic databases and registers on Aug. 26, 2022 to retrieve peer-reviewed literature: Medline (Ovid), Embase (Ovid), CINAHL Complete (EBSCOhost), PsycInfo (EBSCOhost), Web of Science Core Collection (Web of Science), and Cochrane Central Register of Controlled Trials (Ovid). See Additional file 2 for a sample search strategy. We conducted a grey literature search of selected websites and databases from Oct. 27–Nov. 7, 2022. We conducted forward and backward citation chaining from Dec. 1–2, 2022. We updated the searches through an additional round of forward citation chaining conducted on Mar. 31, 2023. Full search strategies can be found in the OSF data repository [55].

### Screening, data extraction, and critical appraisal

We imported all searches into Covidence, an online platform for supporting systematic reviews [56]. Screening, data extraction and critical appraisal were completed in Covidence by two reviewers working independently and blinded to each other’s assessments (AA, SB, RF, TM). See Table 2 for eligibility criteria used in screening. Disagreements were resolved through discussion or by a third reviewer (JL, SB). We used a standardized, pre-piloted form to extract information on study characteristics and findings, including geographical region, study aim, study design, and sample characteristics.

**Table 2:** Eligibility criteria used to screen studies

Inclusion Criteria
**For all studies:**
• Includes findings on the impact of relaxed restrictions on take-home doses of opioid agonist medication for opioid use disorder, either alone or in conjunction with other interventions/exposures, during the Covid-19 pandemic on program effectiveness in opioid agonist treatment • Written in English, French, Spanish, Portuguese, or Italian
**For quantitative component:**
• A randomized or non-randomized study reporting quantitative data **OR** a mixed methods study where the quantitative component can be cleanly extracted • Assesses one or more of the following client outcomes: (1) Retention in treatment, using any quantitative measure; (2) illicit substance use, using any quantitative measure; (3) fatal and non-fatal overdose, using any quantitative measure; (4) client health, using any quantitative measure; (5) client quality of life, using any quantitative measure; (6) client satisfaction with treatment, using any quantitative measure
**For qualitative component:**
• A qualitative study using any qualitative approach (e.g., grounded theory, critical theory, ethnography) **OR** a mixed methods study where the qualitative component can be cleanly extracted • Includes findings on OAT clients’ experiences with relaxed restrictions on take-home doses of OAT during the Covid-19 pandemic
Exclusion Criteria
**For all studies:**
• OAT clients are a subgroup of the study population, but findings specific to this group cannot be extracted; • Take-home doses intended to be supervised remotely or in person (e.g., witnessed daily delivery; take-homes witnessed through videoconferencing systems) • Commentaries, editorials, or letters to the editor, unless original empirical research is presented • Conference abstracts, posters, or slide decks, unless meeting three predefined conditions designed to limit retrieval to relevant studies for which sufficient information can be obtained • The study is a preprint that has become available in peer-reviewed form
**For qualitative component:**
• The study uses quantitative methods (e.g., questionnaires, fixed-choice surveys) to collect qualitative data

Acronyms: *OAT* opioid agonist treatment

We used the Mixed Methods Appraisal Tool (MMAT) version 2018 to appraise study quality and validity [57]. The MMAT is designed specifically for mixed methods systematic reviews. We used the results of the appraisal to assess the strengths and weaknesses of the evidence base and conducted a sensitivity analysis excluding low-quality studies, which we defined as studies meeting fewer than three of five criteria on the MMAT.

### Quantitative synthesis

For the quantitative synthesis, we grouped study findings by outcome to improve comparability. We did not conduct meta-analysis or summarize effect estimates because the diversity of outcome measures precluded calculation of a common effect estimate. Nor was it possible to summarize p-values with the data available. Instead, we synthesized data using vote counting based on direction of effect to answer the question “Is there any evidence of an effect?” [58, 59]. This method is an acceptable alternative to meta-analysis when it is not possible to calculate a standardized estimate of effect, as is often the case in reviews of complex interventions [58–60]. For each outcome, we compared the number of studies showing a beneficial effect with the number showing a harmful effect. As per guidance, we did not take statistical significance or magnitude of effect into account [59].

When a study used more than one measure for the same outcome, we used Boon & Thomson’s revised method [58] to determine the overall direction of effect supported by the study. If the direction of effect was the same (e.g., all beneficial or all harmful) for ≥ 70% of measures, we considered this the overall direction of effect. We recorded the direction as mixed if less than 70% of measures reported a consistent effect direction. We described the results of the synthesis using harvest plots displaying direction of effect, study quality, and sample size [61–63].

We planned to investigate heterogeneity through subgroup analyses based on treatment type (buprenorphine, which had considerably fewer restrictions on take-home doses before the pandemic, versus methadone) and on race and ethnicity. However, formal statistical investigation was not feasible because of insufficient data. Where possible, we explored the effects of treatment type through informal methods; more specifically, through visual inspection of harvest plots in which studies were shaded according to treatment type (methadone vs. buprenorphine).

### Qualitative synthesis

We synthesised qualitative findings using thematic synthesis, which consists of (1) coding studies line-by-line; (2) grouping codes into descriptive themes; and (3) integrating the descriptive themes into analytical themes that address the review question more directly [64]. Thematic synthesis preserves a clear audit trail from data to analytical themes, making it particularly suitable for systematic reviews [65].

**Fig. 1:**
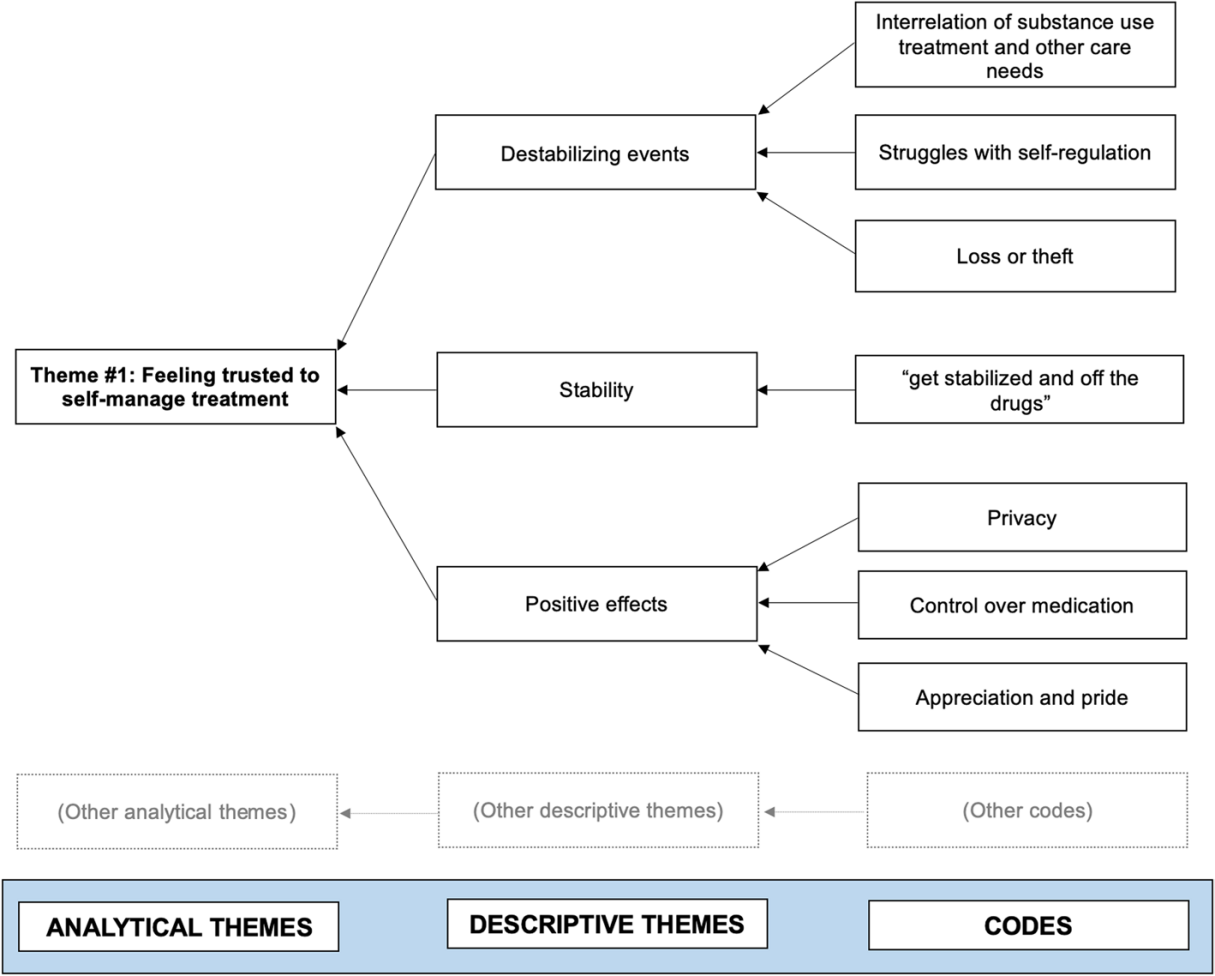
Example of the development of an analytical theme. For visual simplicity, only descriptive themes and codes contributing to Theme #1 are shown

Two members of the research team (AA, SB) coded the same four studies line-by-line in NVivo 1.7 [66]. AA and SB compared and reconciled their coding to create a set of codes and descriptive themes that were used to code/re-code all studies (AA, SB). After coding was completed, AA and SB discussed conceptual links between the descriptive themes and generated analytical themes. These themes were then reviewed with a third member of the research team (EOJ). See Fig. 1 for an illustration of theme development.

### Certainty of evidence

There is no consensus around whether appraising the certainty of the evidence is appropriate in mixed methods reviews, with some organizations supporting this step [67] and others advising against [51]. Methodologists have raised concerns about the use of GRADE and similar methods in mixed methods reviews because of the complexities and uncertainties around incorporating these assessments into the integrated findings of the review [51, 68]. In light of these concerns, we did not formally appraise the certainty of the evidence supporting the qualitative or quantitative findings.

### Integrated analysis

To develop the integrated analysis, we juxtaposed the qualitative and quantitative syntheses and considered how they might complement, explain, or contextualize each other [51]. After drafting the analysis, we discussed our preliminary findings with seven community members with lived experience of OAT to help us assess the credibility of our findings and inform further interpretation.

## Results

**Fig. 2:**
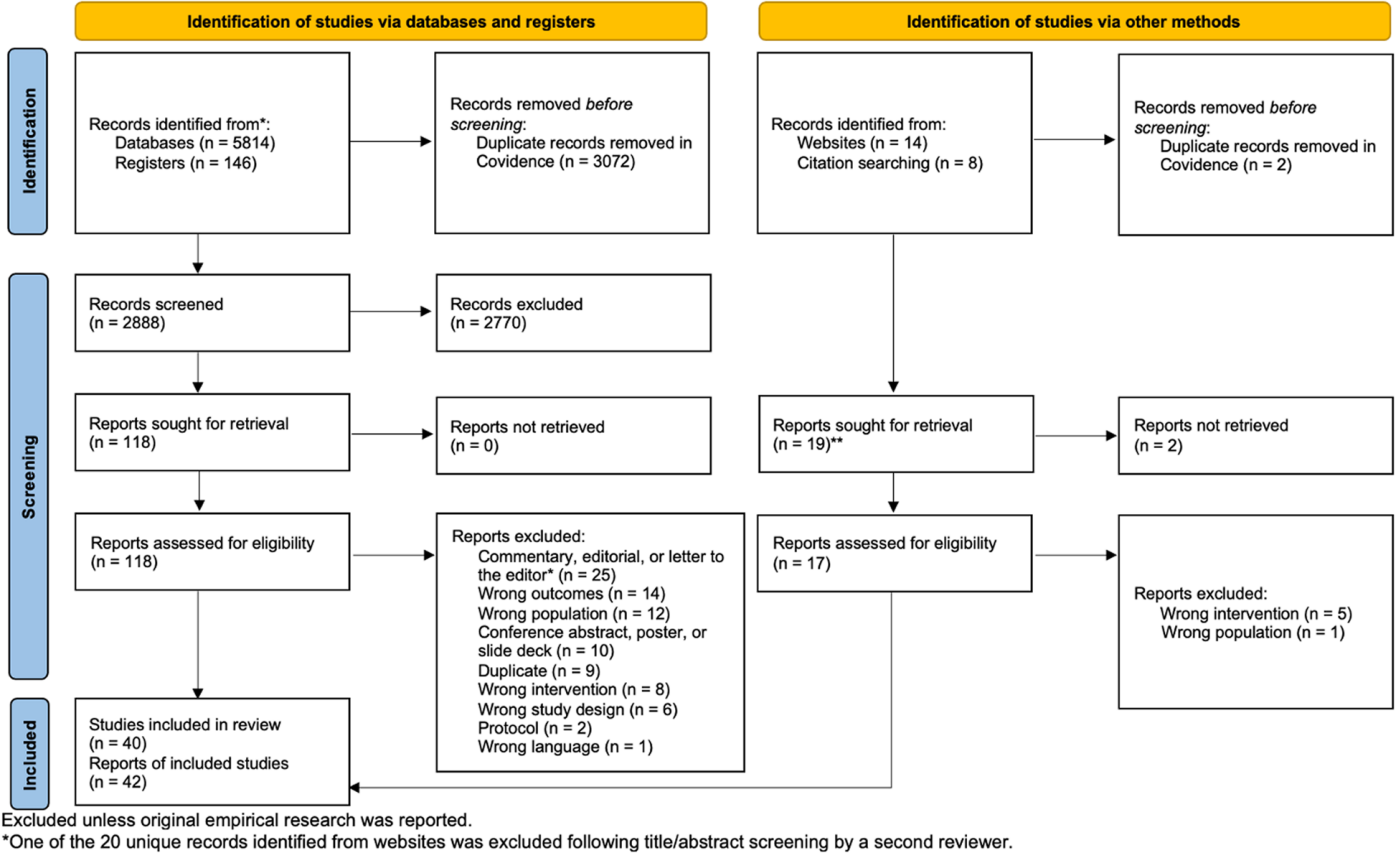
PRISMA diagram

After excluding duplicates, we retrieved 2,888 records from databases and registers and 20 records from citation chaining and the grey literature search. Of these, 42 records (representing 40 studies) met our eligibility criteria and were included in the review [69–110] (hereafter referred to as S1–S40; see Table 3) (Fig. 2).

**Table 3:** Characteristics of included studies

No	Study	Region	Aim^a^	Study Design	Start of Data Collection	End of Data Collection	Q1 Findings (Quant.)	Q2 Findings (Qual.)
S1	Abidogun et al., 2023^b^ [69]	United States	To explore (1) the impact of COVID-19-related changes to methadone treatment, including increased take-home doses, on patients; and (2) the experience of patients with virtual counselor meetings	Qualitative study	Mar. 2021	Jun. 2021	No	Yes
S2	Aldabergenov et al., 2022 [70]	United Kingdom (England)	To investigate methadone- and buprenorphine-related deaths in people prescribed and not prescribed OAT after the first COVID-19 lockdown and compare trends to those observed in prior years	Before-and-after study	Jan. 2016	Jun. 2020	Yes	No
S3	Amram et al., 2021 [71]	United States	To evaluate the effects of a COVID-19-related increase in methadone take-home doses on outcomes for MOUD clients	Before-and-after study	May 2019	Dec. 2020	Yes	No
S4	Bart et al., 2022 [72]	United States	To explore the impact of COVID-19-related changes to methadone take-home doses on drug use	Before-and-after study	Jul. 2019	Jul. 2020	Yes	No
S5	Conway et al., 2023 [73]	Australia	To explore how adaptations to OAT provision “impacted and responded to the risk environments of people receiving OAT during the COVID-19 pandemic” (p. 2)	Qualitative study	Aug. 2020	Dec. 2020	No	Yes
S6	Corace et al., 2022 [74]	Canada	To assess "(1) which patients received additional unsupervised doses during the pandemic; (2) the outcomes of unsupervised dosing [...]; and (3) patients' and prescribers' experiences with changes in OAT care delivery" (p. 2)	Cross-sectional study	Aug. 2020	Sept. 2020	Yes	No
S7	Cunningham et al., 2022 [75]	United States	To understand how COVID-19-related changes in health care policies and health care delivery impacted buprenorphine treatment outcomes	Cohort study	Mar. 2019	Dec. 2020	Yes	No
S8	Ezie et al., 2022 [76]	United States	To investigate changes in medication adherence, illicit substance use, rates of infection, and mortality following SAMHSA's relaxation of take-home guidelines for methadone treatment	Before-and-after study	Dec. 2019	Jun. 2020	Yes	No
S9	Farid et al., 2022 [77]	Bangladesh	NR	Before-and-after study	Jul. 2019	Mar. 2021	Yes	No
S10	Gage et al., 2022 [78]	Online community (Reddit)	"to investigate the lived experience of PWUD during the COVID-19 pandemic" (p. 1505)	Qualitative study	Mar. 2020	Jun. 2020	No	Yes
S11	Garg et al., 2022 [79]	Canada	To investigate the impact of COVID-19, [including the] subsequent change in OAT guidance, on OAT discontinuation" (p. 2)	Time series study	Apr. 2019	Nov. 2020	Yes	No
S12	Gittins et al., 2022 [80]	United Kingdom (England)	To explore over-the-counter and prescription drug misuse among SMS [substance misuse services] clients during COVID-19	Mixed methods (qualitative/cross-sectional)	Aug. 2020	Aug. 2021	No	Yes
S13	Gomes et al., 2022 [81]	Canada	"to evaluate whether increased access to take-home doses of OAT related to pandemic specific guidance was associated with changes in treatment retention and opioid-related harms" (p. 847)	Cohort study	Feb. 2020	NR	Yes	No
S14	Harris et al., 2022 [82]	United States	"to explore how the COVID-19 pandemic impacted MOUD and addiction service experiences." (p. 2)	Qualitative study	Aug. 2020	Oct. 2020	No	Yes
S15	Hoffman et al., 2022 [83]	United States	"to assess patients' responses to the enhanced access to take-home methadone" (p.2)	Mixed methods (qualitative/before-and-after)	Sept. 2019	Dec. 2020	Yes	Yes
S16	Javakhishvili et al., 2021 [84]	Western Georgia (Eurasia)	To study treatment satisfaction and quality of life among people in opioid substitution therapy (OST) programs in western Georgia [during the COVID-19 pandemic]	Mixed methods (qualitative/cross-sectional)	NR; data collection "during pandemic"	NR; data collection "during pandemic"	Yes	Yes
S17	Joseph et al., 2021 [85]	United States	The original research presented in this commentary was conducted "to ascertain outcomes" of new approach to take-home dosing following SAMHSA's relaxation of take-home guidelines for methadone treatment	Before-and-after study	Jan. 2020	May 2020	Yes	No
S18	Kesten et al., 2021 [86]	United Kingdom	To understand how people who inject drugs experienced COVID-19-related public health measures and changes to opioid substitution treatment and harm reduction services	Qualitative study	Jun. 2020	Aug. 2020	No	Yes
S19	Krawczyk et al., 2021 [87]	Online community (Reddit)	To explore views on the impact of the COVID-19 pandemic on various aspects of treatment for opioid use disorder	Qualitative study	Mar. 2020	NR	No	Yes
S20	Levander et al., 2021 [88]	United States	To investigate patients' perceptions of the impact of COVID-19-related changes to take-home methadone policies and to investigate how these changes affected treatment access, recovery, and mental health support for rural patients	Qualitative study	Aug. 2020	Jan. 2021	No	Yes
S21	Liddell et al., 2021 [89]	United Kingdom (Scotland)	"to provide a baseline of current MAT [medication-assisted treatment] provision, prior to implementation [of new treatment standards], from the perspective of people currently in treatment" (p. 6). [Includes experiences with increased take-home doses during the pandemic]	Mixed methods (qualitative/cross-sectional)	Dec. 2020	May 2021	No	Yes
S22	Lintzeris et al., 2022 [90]	Australia	To describe COVID-19-related changes to OAT service delivery and to examine changes in patient outcomes following the implementation of the changes	Before-and-after study	Dec. 2019	Sept. 2020	Yes	No
S23	May et al., 2022 [91]	United Kingdom	To "[investigate] the longer-term impacts of the pandemic on the health and wellbeing [...] of PWID, as well as their experiences of treatment changes from the perspectives of both PWID and service providers" (p. 2)	Qualitative study	May 2021	Sep. 2021	No	Yes
S24	Meyerson et al., 2022 [92]	United States	"To understand patient experience of federal regulatory changes governing methadone and buprenorphine (MOUD) access in Arizona during the COVID-19 pandemic" (p. 1)	Qualitative study	Aug. 2021	Oct. 2021	No	Yes
S25	Morin et al., 2021 [93]	Canada	"to present a Canadian perspective on increased fentanyl positive urine drug screen results among OAT patients during the COVID-19 pandemic." (p. 2)	Time series study	Jan. 2020	Sept. 2020	Yes	No
S26	Nguyen et al., 2021^c^ [94]	United States	"to understand the impact of the expanded eligibility for take-home MOUD dosing, including benefits and unintended consequences" (p. 3)	Mixed methods (qualitative/before-and-after and cohort data)	Jan. 2019	Dec. 2020	Yes	No^b^
S27	Nobles et al., 2021 [95]	Online community (Reddit)	To address the knowledge gap around "the perspectives and experiences of OTP [opioid treatment program] patients during the COVID-19 pandemic [...] we qualitatively examine self-reported impacts to the delivery of MMT." (p. 2135)	Qualitative study	Jan. 2020	Sept. 2020	No	Yes
S28	Parkes et al., 2021 [96]	United Kingdom (Scotland)	To explore how program staff and PWLLE have experienced COVID-19 related changes to services for people experiencing homelessness and problem substance use	Qualitative study	Apr. 2020	Aug. 2020	No	Yes
S29	Pilarinos et al., 2022 [97]	Canada	"to identify policy related factors that can be addressed to improve OAT experiences and outcomes among young people, and we provide new insights into how OAT programming can be optimized to meet young peoples' needs and goals." (p. 2). [Includes experiences with COVID-19-related changes to take-home dosing]	Qualitative study	Jan. 2018	Aug. 2020	No	Yes
S30	Rosic et al., 2022 [98]	Canada	"1. To determine whether opioid use increased, decreased, or remained unchanged during the COVID-19 pandemic for patients already enrolled in MAT; 2. To explore factors associated with a change in the percentage of opioid-positive urine drug screens (UDSs) for patients followed both before and during the COVID-19 pandemic." (p. e258)	Before-and-after study	Jun. 2019	Nov. 2020	Yes	No
S31	Roy et al., 2023 [99]	United States	To evaluate "national changes in buprenorphine access as a result of COVID-19-related prescribing guideline changes up to one-year post-initial-pandemic period" (p. 2)	Time series study	Feb. 2019	Apr. 2021	Yes	No
S32	Russell et al., 2021 [100]	Canada	"to understand how service disruptions during COVID-19 may have affected PWUD" (p. 2)	Qualitative study	May 2020	Jul. 2020	No	Yes
S33	Schofield et al., 2022 [101]	United Kingdom (Scotland)	To explore "the impacts of COVID-19 related changes on the availability and uptake of health and care services, particularly harm reduction, treatment, recovery, and general healthcare services, among PWUD in Scotland during the pandemic" (p. 2)	Qualitative study	May 2020	Nov. 2020	No	Yes
S34	Scott et al., 2023 [102]	United Kingdom (England)	To investigate how people with OUD experienced changes to substance use treatment during COVID-19 and to explore their views on improving OAT delivery	Qualitative study	NR	NR	No	Yes
S35	Suen et al., 2022/Wyatt et al., 2022 [104]	United States	"to describe the MOUD treatment experiences of patients and providers at an OTP [opioid treatment program] in San Francisco, California, to inform [post-COVID-19] research and policy" (p. 1148)	Qualitative study	Aug. 2020	Nov. 2020	No	Yes
S36	University of Bath et al., 2020, 2021 [106]	England	"to understand how people in receipt of OST [opioid substitution treatment] in rural areas have experienced the pandemic changes [to treatment]." (p. 2)	Qualitative study	NR	Mar. 2021	No	Yes
S37	Vicknasingam et al., 2021 [107]	Malaysia	To evaluate how people who use drugs and service providers adapted to and coped with COVID-19-related public health measures and associated changes to treatment	Qualitative study with before-and-after quantitative data	Dec. 2019	Aug. 2020	Yes	Yes
S38	Walters et al., 2022 [108]	United States	To examine how COVID-19 and COVID-19 mitigation strategies "affected the lives of people who use drugs in relation to MOUD" (p. 1145)	Qualitative study	Jun. 2020	Oct. 2020	No	Yes
S39	Watson et al., 2022 [109]	United States	"[to investigate] how individuals with OUD understood and navigated treatment and their personal recoveries during the COVID-19 pandemic" (p. 2)	Qualitative study	Sept. 2020	Jan. 2021	No	Yes
S40	Zhen-Duan et al., 2022 [110]	United States	"to understand (1) how the COVID-19 pandemic impacted low-income individuals with SUD [substance use disorder] and (2) how people adjusted to SUD treatment changes during stay-at-home orders in NYC [New York City]" (p. 1105)	Qualitative study	Apr. 2020	Jun. 2020	No	Yes

^a^Acronyms: *MMT* methadone maintenance treatment, *MOUD* medication for opioid use disorder, *NR* not reported, *OAT* opioid agonist treatment, *OUD* opioid use disorder, *PWID* people who inject drugs, *PWLLE* people with lived and living experience [of substance use], *PWUD* people who use drugs, *SAMHSA* Substance Abuse and Mental Health Administration

^b^Qualitative findings were extracted from a preprint version of this manuscript. Comparison with the peer-reviewed publication showed no appreciable changes to the data extracted for this review

^c^Qualitative findings from this mixed-methods preprint were later published in peer-reviewed form (Suen et al., 2022/Wyatt et al., 2022) and were therefore not extracted from the preprint

### Study characteristics

Most studies were from the United States (16/40), the United Kingdom (9/40), or Canada (7/40). Twenty-four studies included participants on a variety of OAT medications. Fourteen focused exclusively on methadone clients and two were limited to buprenorphine clients. For additional details on study design and participant characteristics, see Tables 3 and 4.

**Table 4:** Characteristics of participants in included studies

No	Study	Sample^a^	No. of OAT Clients in Sample	Opioid Medication(s) Used	Age^b^	Sex	Race and Ethnicity^e^
S1	Abidogun et al., 2023 [69]	28 clients from a community-based opioid treatment program serving a low-income population in Baltimore, Maryland	28	Methadone	50 (10)	Female: 43% Male: 57%	White: 39% Black/African American: 57% American Indian: 4%
S2	Aldabergenov et al., 2022 [70]	529 deceased adults prescribed and not prescribed OAT treatment for opioid use disorder in England	NR	Buprenorphine, methadone	NR	Female: NR Male: NR	NR
S3	Amram et al., 2021 [71]	183 MOUD clients at an opioid treatment program in Spokane County, Washington	183	Methadone	41 (median), 32–51 (IQR)	Female: 58% Male: 42%	Non-Hispanic White: 73% Other: 18%
S4	Bart et al., 2022 [72]	613 clients at the Hennepin Healthcare Addiction Medicine opioid treatment program in Minnesota	613	Methadone	49 (14)	Female: 49% Male: NR	Caucasian: 46% Black: 23% American Indian: 15% Asian: 9% Latinx: < 1%
S5	Conway et al., 2023 [73]	40 OAT clients and 28 OAT providers in Australia	40	Buprenorphine, methadone	NR	NR	NR
S6	Corace et al., 2022 [74]	402 OAT clients prescribed OAT and 100 OAT prescribers in Ontario.^d^	402	Buprenorphine, methadone, slow-release oral morphine	18–59 (range)	Female: 44% Male: 54% Trans and/or GE: 2%	White– European or North American: 78% Black – African, Caribbean, or North American: 11% First Nations, Inuit, or Metis: 7% Asian – East or South East: 2% Latin American: 1% Mixed heritage: < 1% Prefer not to respond: < 1%
S7	Cunningham et al., 2022 [75]	107 people referred for buprenorphine treatment at Montefiore Buprenorphine Treatment Network in the Bronx (NY, USA)	81	Buprenorphine	46 (14)	Female: 33% Male: NR	Hispanic: 52% Non-Hispanic Black: 20% Non-Hispanic White: 18% Non-Hispanic other or unknown: 10%
S8	Ezie et al., 2022 [76]	129 clients at a methadone maintenance treatment program in New York	129	Methadone	66 (median), 32–79 (range)	Female: 1% Male: 99%	Non-Hispanic Black/African American: 40% Non-Hispanic White: 28% Hispanic or Latino: 25% American Indian/Alaska Native: 2% Unknown: 5%
S9	Farid et al., 2022 [77]	PWID receiving opioid substitution treatment at 35 centers in Bangladesh	NR	Methadone	NR	Female: NR Male: NR	NR
S10	Gage et al., 2022 [78]	100 posters on four Reddit subforums related to substance use	NR	Buprenorphine, methadone	16 (7)^c^	Female: NR Male: NR	NR
S11	Garg et al., 2022 [79]	63,941 clients receiving methadone or buprenorphine/naloxone in Ontario	63,941	Buprenorphine, methadone	NR	Female: NR Male: NR	NR
S12	Gittins et al., 2022 [80]	56 clients receiving substance use care at two community treatment centres/providers in England	35	Buprenorphine, methadone	39 (mean), 18–61 (range)	Female: 41% Male: 59%	White—British: 95% White – Irish: 4% White – Other: 2%
S13	Gomes et al., 2022 [81]	21,297 people receiving OAT in Ontario	21,297	Buprenorphine, methadone	NR [only subgroup data reported]	NR [only subgroup data reported]	NR
S14	Harris et al., 2022 [82]	20 Boston site participants from a parent study (REBOOT) on preventing opioid overdose	14	Buprenorphine, methadone	42 (mean), 27–61 (range)	Female: 45% Male: 50% Trans and/or GE: 5%	White: 80% Other or more than one race: 20%
S15	Hoffman et al., 2022 [83]	377 methadone clients at two opioid treatment programs serving five Southern Oregon rural counties	377	Methadone	40 (11)	Female: 49% Male: 51%	Non-Hispanic White: 93%
S16	Javakhishvili et al., 2021 [84]	100–668 clients from OST institutions in Western Georgia (Eurasia)	Quant: 668, Qual.: 10	Buprenorphine, methadone	43 (median) [quant. participants], 48 (6) [qual. participants]	Female: 10% Male: 90%	NR
S17	Joseph et al., 2021 [85]	> 3,600 opioid treatment program clients at five clinics in the Bronx	> 3,600	Methadone	NR	Female: NR Male: NR	NR
S18	Kesten et al., 2021 [86]	28 people who use drugs in Bristol, England	23	Buprenorphine, methadone	25–29: 7% 30–34: 14% 35–39: 36% 40–44: 18% 45–49: 11% 50–54: 14%	Female: 32% Male: 68%	NR
S19	Krawczyk et al., 2021 [87]	Posters on the subreddits r/Opiates and r/OpiatesRecovery	NR	Buprenorphine, methadone	NR	Female: NR Male: NR	NR
S20	Levander et al., 2021 [88]	46 clients at three rural opioid treatment programs in Oregon	46	Methadone	44 (13)	Female: 50% Male: 50%	White: 96% American Indian/Alaska Native: 13% Hispanic/Latinx: 4%
S21	Liddell et al., 2021 [89]	95 MAT clients from six health boards across Scotland	90	Buprenorphine, methadone	24–34: 25% 35–44: 53% 45–54: 16% 55–64: 5% Missing: 1%	Female: 43% Male: 56%	White – Scottish: 96% White – British: 6% White – English: 3%
S22	Lintzeris et al., 2022 [90]	429 clients enrolled on OAT at three public treatment service locations in Sydney	429	Buprenorphine, methadone	43 (10)	Female: 33% Male: NR	NR
S23	May et al., 2022 [91]	19 PWID recruited through drug and homelessness services in London and Bristol	NR	Methadone	40 (mean), 24–49 (range)	Female: 53% Male: 47%	White British: 68% Black or Black British Caribbean: 11% White and Black Caribbean: 11% White Other: 11%
S24	Meyerson et al., 2022 [92]	131 MOUD clients from 29 different providers in rural and urban communities across Arizona	131	Buprenorphine, methadone	38 (11)	Female: 38% Male: 71% Trans and/or GE: 2%	White: 68% Hispanic: 24% Black: 3% Native American: 3% Asian: 2%
S25	Morin et al., 2021 [93]	14,669 clients from 67 OAT clinics in Ontario	14,669	Buprenorphine, methadone^g^	NR	Female: NR Male: NR	NR
S26	Nguyen et al., 2021^f^ [94]	506 clients at a hospital-affiliated opioid treatment program in San Francisco, California	506	Methadone	48 (11)	Female: NR Male: 77%	White: 51% Black/African American: 32% Hispanic: 10% Other: 7%
S27	Nobles et al., 2021 [95]	179 posters on the subreddit r/methadone	NR	Methadone	NR	Female: NR Male: NR	NR
S28	Parkes et al., 2021 [96]	10 people with lived/living experience of homelessness who used services at the Wellbeing Centre, as well as staff and stakeholders	NR	Methadone^g^	NR	Female: 20% Male: 80%	NR
S29	Pilarinos et al., 2022 [97]	56 young current or former OAT clients in Vancouver	NR	Buprenorphine, methadone, slow-release oral morphine^g^	14–24 (range)	Female: 32% Male: 64% Trans and/or GE: 4%	White: 75% Indigenous: 23% Asian-Canadian: 9% African-Canadian: 5% Declined to answer: 4%
S30	Rosic et al., 2022 [98]	629 OAT clients from 31 clinics in Ontario	629	Buprenorphine, methadone	40 (11)	Female: NR Male: 50%	NR
S31	Roy et al., 2023 [99]	Individuals prescribed buprenorphine in the U.S. between Feb. 2019 and Apr. 2021	Time point 1: 1,269,651 Time point 2: 814,013 Time point 3: 1,329,502	Buprenorphine	Time point 1: 41 (12) Time point 2: 42 (13) Time point 3: 42 (12)	Varied; 43–44%	NR
S32	Russell et al., 2021 [100]	196 people who use drugs from across Canada	72	Buprenorphine, methadone, “intravenous OAT”	41 (11)	Female: 41% Male: 56% Trans and/or GE: 4%	White: 59% Indigenous: 30% Other: 11%
S33	Schofield et al., 2022 [101]	29 people who use drugs recruited from a hostel/shelter, a stabilization and housing service, a harm reduction service, and a peer-led recovery community in Scotland	NR	Buprenorphine, methadone^g^	28–56 (range)	Female: 45% Male: 55%	NR
S34	Scott et al., 2023 [102]	27 people receiving OAT at a community addictions centre in London	27	Buprenorphine, methadone	47 (NR)	Female: 19% Male: 82%	White British: 52% Black British: 15% Other: 33%
S35	Suen et al., 2022/Wyatt et al., 2022 [104]	20 MOUD patients and 10 providers at one OTP in San Francisco, California	20	Buprenorphine, methadone	51 (median), 41–60 (IQR)	Female: 47% Male: 53%	Black/African American: 47% Hispanic/Latinx: 26% White: 26% Native American/American Indian: 11%
S36	University of Bath et al., 2020, [105]	15 people receiving OST in rural villages and towns in Somerset, Wiltshire, and Suffolk	15	Methadone^g^	43 (mean), 31–56 (range)	Female: 53% Male: 47%	NR
S37	Vicknasingam et al., 2021 [107]	Methadone maintenance treatment (MMT) clients and personnel at MMT programs, HIV clinics, and NGO services in the Malaysian states of Penang, Kelantan, Selangor, and Melaka	Quant.: 74, Qual.: 9	Methadone	NR	Female: NR Male: NR	NR
S38	Walters et al., 2022 [108]	37 people who use drugs recruited from the Northeast US; 18 MOUD providers, clinic staff, and regulatory officials	NR	Buprenorphine, methadone	NR	Female: NR Male: NR	NR
S39	Watson et al., 2022 [109]	25 people referred to MOUD in Chicago, Illinois, within the year prior to or after the start of the COVID-19 pandemic	20	Buprenorphine, methadone	57 (mean), 48–74 (range)	Female: 76% Male: 24%	African American: 96% Hispanic/Latino: 4%
S40	Zhen-Duan et al., 2022 [110]	20 adults enrolled in Medicaid and receiving outpatient SUD treatment (e.g., medication, counseling) in NYC	13	Buprenorphine, methadone	52 (13)	Female: 20% Male: 80%	Black non-Latinx: 25% Asian non-Latinx: 20% Black Latinx: 10% White Latinx: 10% Multiracial non-Latinx: 5% Multiracial Latinx: 5% Latinx, no race selected: 25%

^a^Acronyms: *GE* gender-expansive, *IQR* interquartile range, *NR* not reported, *SD* standard deviation

^b^Ages presented as mean (SD), unless otherwise specified. All ages are rounded to the nearest year

^c^Based on 53 posts reporting age

^d^Prescriber data excluded from sample characteristics

^e^Race and ethnicity were initially extracted in dichotomized form (White/Non-White) to facilitate subgroup analysis. As subgroup analysis was not possible, one reviewer (AA) subsequently extracted a more detailed breakdown of the ‘Non-White’ category using the terms used in the original studies. All figures are rounded to the nearest percent. Some figures sum to more than 100% because of rounding and/or selection of multiple race and ethnicity categories

^f^Qualitative findings from this mixed-methods preprint were later published in peer-reviewed form (Suen et al., 2022/Wyatt et al., 2022) and were therefore not extracted. Sample characteristics in this table are based on participants in the quantitative analysis

^g^Inferred from type of treatment facility, general description of treatment, or participant quotes; may not be exhaustive list of participants’ medications

Eighteen studies contributed data to the quantitative synthesis. As specified in our review protocol, we included studies in which the relaxation of restrictions on take-home doses formed part of a broader intervention or exposure. Other pandemic-related changes to OAT described in the quantitative studies included increased use of telehealth and virtual care (S2, S6, S7, S11, S13, S22, S30, S31), reduced in-person appointments (S6, S7, S11, S13, S15–17, S22), cessation or reduced frequency of urine testing (S2, S6, S11, S17, S22, S37), home delivery of medication for clients who were self-isolating and/or at high risk (S7, S22, S30), rapid or remote protocols for OAT induction (S2, S30, S31), and increased naloxone provision (S7, S22). Of the 18 studies, nine were intended to assess only the impact of changes to take-home policies. Five of these studies (S3, S4, S13, S15, S22) used methods to control for the impact of co-exposures or other factors associated with the receipt of take-home doses (e.g., regression modelling) in their analysis. Six studies defined their intervention of interest as pandemic-related changes to OAT, including, but not limited to, increased take-home doses. Two studies defined their exposure/intervention as the pandemic together with associated changes to OAT.

Twenty-five studies contributed to the qualitative synthesis. Three focused exclusively on OAT clients’ experiences with take-home during the COVID-19 pandemic. Many were designed to explore participants’ experiences with any and all pandemic-related changes to OAT (15/25). A smaller number explored how people who use drugs experienced life during the pandemic (7/25). Though all studies met our inclusion criteria, some contributed little data to the synthesis.

### Quantitative synthesis

**Fig. 3:**
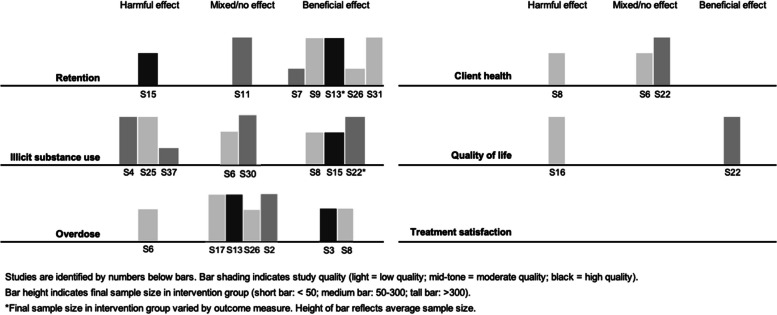
Harvest plots showing results of synthesis by direction of effect

Visual inspection of harvest plots (see Fig. 3) suggested an association between take-home doses and improved retention, but showed no clear evidence of an association with overdose or illicit substance use. The small number of studies reporting client health or quality of life precluded meaningful synthesis. We did not identify any studies reporting treatment satisfaction. Brief narrative summaries are provided below.

#### Retention

Seven studies reported measures of retention, including one finding a negative direction of effect (S15), one with mixed direction of effect (S11), and five supporting a positive direction of effect (S7, S9, S13, S26, S31). See Table 5. Two were high-quality (S13, S15), two were moderate-quality (S7, S11), and three were low-quality (S9, S26, S31). Our main concerns about the quality of studies contributing to this outcome were failure to account for confounding, unplanned co-interventions, and generalizability (Table 6).

**Table 5:** Studies reporting measures of retention

Study	Measure	Control Group	Intervention Group	Statistical Test or Model	*p*-value	Estimate of Effect	Direction of Effect	Overall Effect Direction
(S7) Cunningham et al., 2022 [75]	Retention in treatment at 90 days^a^	42.9%^b^	68.0%^c^	Chi square or Fisher exact test	< 0.05	NR	Favours intervention	Positive
(S9) Farid et al., 2022 [77]	“Retention”	68.1%^d^	(a) 72.9% (b) 82.7% (c) 87.3%^e^	NR	NR	NR	Favours intervention	Positive
(S11) Garg et al., 2022 [79]	Immediate change^f^ in weekly prevalence of treatment discontinuation following intervention among clients stable on OAT	NA	NA	Autoregressive integrated moving average (ARIMA) model	0.93	Step change: -0.01% (95% CI -0.14–0.12%)	Favours intervention	Mixed
	Gradual change^g^ in weekly prevalence of treatment discontinuation following intervention among clients stable on OAT	NA	NA	Autoregressive integrated moving average (ARIMA) model	0.72	Slope change: 0.00% (95% CI -0.01–0.02%)	No difference	
	Immediate change^f^ in weekly prevalence of treatment discontinuation following intervention among clients not stable on OAT	NA	NA	Autoregressive integrated moving average (ARIMA) model	0.82	Step change: -0.31% (-3.04–2.43%)	Favours intervention	
	Gradual change^g^ in weekly prevalence of treatment discontinuation following intervention among clients not stable on OAT	NA	NA	Autoregressive integrated moving average (ARIMA) model	0.63	Slope change: 0.04% (95% CI: -0.12–0.20%)	Favours control	
(S13) Gomes et al., 2022 [81]	OAT discontinuation^h^ among people receiving daily methadone at baseline	63.6% per person-year^i^	51.0% per person-year^j^	Cox proportional-hazards model	< 0.05*	Weighted HR: 0.80 (95% CI: 0.72–0.90)	Favours intervention	Positive
	OAT discontinuation^h^ among people receiving 5–6 take-home doses of methadone at baseline	19.6% per person-year^i^	14.1% per person-year^j^	Cox proportional-hazards model	< 0.05*	Weighted HR: 0.72 (95% CI 0.62–0.84)	Favours intervention	
	OAT discontinuation^h^ among people receiving daily buprenorphine/naloxone at baseline	93.2% per person-year^i^	85.1% per person-year^j^	Cox proportional-hazards model	≥ 0.05*	Weighted HR: 0.91 (95% CI 0.68–1.22)	Favours intervention	
	OAT discontinuation^h^ among people receiving 5–6 take-home doses of buprenorphine/naloxone at baseline	30.8% per person-year^i^	26.0% per person-year^j^	Cox proportional-hazards model	≥ 0.05*	Weighted HR: 0.85 (95% CI 0.70–1.01)	Favours intervention	
(S15) Hoffman et al., 2022 [83]	Treatment discontinuation among people in treatment < 90 days	13%^k^	26%^l^	Wilcoxon rank sum test; Pearson's Chi-squared test	0.047	NR	Favours control	Negative
	Treatment discontinuation among people in treatment 90–180 days	9.4%^k^	19%^l^	Wilcoxon rank sum test; Pearson's Chi-squared test	0.090	NR	Favours control	
	Treatment discontinuation among people in treatment > 180 days	11%^k^	12%^l^	Wilcoxon rank sum test; Pearson's Chi-squared test	0.7	NR	Favours control	
	Odds of treatment discontinuation per percentage point in take-home dosing above expected^m^	NR	NR	Random effects logistic regression model	0.003	Adjusted OR: 0.97 (95% CI 0.95, 0.99)	Favours intervention	
(S26) Nguyen et al., 2021 [94]	60-day retention among new intakes	63%^k^	69%^l^	Two-tailed t-test	0.26	NR	Favours intervention	Positive
(S31) Roy et al., 2023 [99]	Treatment disruptions among stably treated clients^n^ at 1 week post-initial pandemic period	1.3%^k^	NR	Segmented regression interrupted time series model	< 0.05	Relative change from baseline trend: (a) Disruptions ≥ 7 days: -12.6 (95% CI: -16.6,-8.5) (b) Disruptions ≥ 14 days: -9.7 (95% CI: -15.1,-4.3) (c) Disruptions ≥ 28 days: -11.6 (95% CI: -14.7,-8.5)	Favours intervention	Positive
	Treatment disruptions among stably treated clients^n^ at 26 weeks post-initial pandemic period	1.0%^k^	NR	Segmented regression interrupted time series model	< 0.05	Relative change from baseline trend: (a) Disruptions ≥ 7 days: -17.0 (95% CI: -19.4,-14.6) (b) Disruptions ≥ 14 days: -10.2 (95% CI: -15.7,-4.8) (c) Disruptions ≥ 28 days: -15.5 (95% CI: -18.9,-12.1)	Favours intervention	
	Treatment disruptions among stably treated clients^n^ at 52 weeks post-initial pandemic period	0.6%^k^	NR	Segmented regression interrupted time series model	< 0.05	Relative change from baseline trend: (a) Disruptions ≥ 7 days: -21.6 (95% CI: -25.6,-17.7) (b) Disruptions ≥ 14 days: -10.8 (95% CI:-16.3,-5.3) (c) Disruptions ≥ 28 days: -27.3 (95% CI:-33.0,-21.6)	Favours intervention	

Where adjusted and unadjusted effect estimates were reported, we present adjusted estimates. Where weighted and unweighted effect estimates were reported, we present weighted estimates. In no case did this change the estimated direction of effect

*Acronyms*: *HR* hazard ratio, *NR* not reported, *OR* odds ratio, *RR* relative risk

^*^Not reported in the original study; inferred or calculated by authors

^a^Retention defined as active buprenorphine prescription at least 90 days after treatment initiation

^b^Control group: OAT clients initiating treatment after referral before pandemic

^c^Intervention group: OAT clients initiating treatment after referral during pandemic

^d^Control group: OAT clients from Jul.–Dec. 2019

^e^Intervention groups: OAT clients from (a) Jan.-Jun. 2020, (b) Jul.-Dec. 2020, and (c) Jan.-Mar. 2021

^f^The step transfer function was used to test for immediate change

^g^The ramp transfer function used to test for gradual change

^h^OAT discontinuation defined as a gap in therapy exceeding 14 days

^i^Control group: OAT clients with no change in take-home doses during pandemic

^j^Intervention group: OAT clients with increased take-home doses during pandemic

^k^Control group: OAT clients pre-pandemic

^l^Intervention group: OAT clients post-pandemic

^m^Analysis limited to OAT clients with at least three months of pre-pandemic data and one month of post-pandemic data

^n^ “Stable clients” defined as clients with six months or more of buprenorphine prescriptions without a treatment disruption. “Treatment disruptions” defined as gaps of 28 days

**Table 6:** Critical appraisal of quantitative studies reporting retention

No	Study	MMAT Section 3^a^ for quantitative non-randomized studies				
		1	2	3	4	5
		Are the participants representative of the target population?	Are measurements appropriate regarding both the outcome and intervention (or exposure)?	Are there complete outcome data?	Are the confounders accounted for in the design and analysis?^b^	During the study period, is the intervention administered (or exposure occurred) as intended?^b^
S7	Cunningham et al., 2022 [75]	No	Yes	Yes	No	Yes
S9	Farid et al., 2022 [77]	Yes	No	Can't tell	No	No
S11	Garg et al., 2022 [79]	Yes	Yes	Yes	No	No
S13	Gomes et al., 2022 [81]	Yes	Yes	Yes	Yes	Yes
S15	Hoffman et al., 2022 [83]	No	Yes	Yes	Yes	Yes
S26	Nguyen et al., 2021 [94]	No	Yes	Yes	No	No
S31	Roy et al., 2023 [99]	Yes	Yes	Can't tell	No	No
# meeting quality criteria		4/7	6/7	5/7	2/7	3/7

^a^The MMAT (Mixed Methods Appraisal Tool) Qualitative Checklist is designed specifically for mixed methods systematic reviews (Hong et al., 2018). It consists of five sections specific to various study designs, each with five quality criteria. All quantitative studies included in this review, including quantitative components of mixed-methods studies, were appraised under *Sect. 3: Quantitative non-randomized studies*

^b^This review included studies in which the intervention of interest (relaxed restrictions on take-home doses) formed part of a broader intervention (e.g., pandemic-related changes to OAT treatment). To increase the relevancy of the quality assessments, we interpreted questions 4 and 5 relevant to the research question posed in this review

##### Negative direction

A before-and-after study (S15) found that treatment discontinuation increased following the relaxation of restrictions on take-home doses, regardless of time in treatment. However, logistic regression showed that the odds of treatment discontinuation decreased with each additional take-home dose.

##### Mixed direction

The overall direction of effect was mixed in a study using statistical modelling to test for changes in OAT discontinuation after pandemic-related treatment changes (S11). Though there was an immediate decrease in treatment discontinuation for all clients, tests for gradual changes showed no change among stable clients and a negative trend for non-stable clients.

##### Positive direction

Five studies reported a positive direction of effect (S7, S9, S13, S26, S31). A cohort study of buprenorphine clients (S7) found that clients referred to treatment during the pandemic, when prescription durations increased, had a higher rate of retention at 90 days than clients referred prior to the pandemic. Another cohort study (S13) assessed the risk of OAT discontinuation in a sample stratified by treatment type and number of take-home doses at baseline. In all four subgroups, clients who received additional take-home doses during COVID-19 had a lower risk of treatment discontinuation. Two before-and-after studies reported increased retention following the relaxation of restrictions on take-home doses (S9, S26), and a time series study using data on buprenorphine prescriptions in the United States (S31) reported a reduction in treatment disruptions of 28 days or more during the pandemic.

#### Illicit substance use

Eight studies reported measures of illicit substance use, including three supporting a negative direction of effect (S4, S25, S37), two with mixed direction of effect (S6, S30), and three finding a positive direction of effect (S8, S15, S22). See Table 7. One study was high-quality (S15), four were moderate-quality (S4, S22, S30, S37), and three were low-quality (S6, S8, S25). Most studies supporting this outcome were downgraded for concerns about unplanned co-interventions, failure to account for confounders, and generalizability (see Table 8).

**Table 7:** Studies reporting measures of illicit substance use

Study	Measure	Control Group	Intervention Group	Statistical Test or Model	*p*-value	Estimate of Effect	Direction of Effect	Overall Effect Direction
(S4) Bart et al., 2022 [72]	Urine test positive for opiates without confirmed prescription	14%^a^	22%^b^	NR	< 0.001	NR	Favours control	Negative
	Urine test positive for amphetamines without confirmed prescription	10%^a^	16%^b^	NR	< 0.001	NR	Favours control	
	Urine test positive for barbiturates without confirmed prescription	0.2%^a^	0.3%^b^	NR	p ≥ 0.001	NR	Favours control	
	Urine test positive for benzodiazepines without confirmed prescription	6.3%^a^	11%^b^	NR	< 0.001	NR	Favours control	
	Urine test positive for cocaine without confirmed prescription	11%^a^	12%^b^	NR	p ≥ 0.001	NR	Favours control	
	Urine test positive for oxycodone without confirmed prescription	2.6%^a^	3.2%^b^	NR	p ≥ 0.001	NR	Favours control	
	Urine test positive for opioids (opiates or oxycodone) without confirmed prescription	NR^a^	NR^b^	Generalized linear mixed model	NR	OR: 2.34 (95% CI 1.78–3.07)	Favours control	
	Urine test positive for non-opioids without confirmed prescription	NR^a^	NR^b^	Generalized linear mixed model	NR	OR: 2.48 (95% CI 1.89–3.25)	Favours control	
	Proportion of drug tests positive for opioids among clients with 1–2 take-home doses/week	0.435^c^	0.202^d^	Generalized linear mixed model	NR	NR	Favours intervention	
	Proportion drug tests positive for opioids among clients with 3–5 take-home doses/week	0.187^c^	0.226^d^	Generalized linear mixed model	NR	NR	Favours control	
	Proportion of drug tests positive for opioids among clients with 6 take-home doses/week	0.060^c^	0.121^d^	Generalized linear mixed model	NR	NR	Favours control	
	Proportion of drug tests positive for opioids among clients with > 6 take-home doses/week	0.027^c^	0.036^d^	Generalized linear mixed model	NR	NR	Favours control	
	Proportion of drug tests positive for non-opioids among clients with 1–2 take-home doses/week	0.587^c^	0.398^d^	Generalized linear mixed model	NR	NR	Favours intervention	
	Proportion drug tests positive for non-opioids among clients with 3–5 take-home doses/week	0.187^c^	0.377^d^	Generalized linear mixed model	NR	NR	Favours control	
	Proportion of drug tests positive for non-opioids among clients with 6 take-home doses/week	0.119^c^	0.161^d^	Generalized linear mixed model	NR	NR	Favours control	
	Proportion of drug tests positive for non-opioids among clients with > 6 take-home doses/week	0.049^c^	0.040^d^	Generalized linear mixed model	NR	NR	Favours intervention	
(S6) Corace et al., 2022 [74]	OAT clients reporting increase in opioid use "since COVID-19 (March 2020)"	46%^e^	28%^f^	NR	NR	NR	Favours intervention	Mixed
	OAT clients reporting decrease in opioid use "since COVID-19 (March 2020)"	21%^e^	14%^f^	NR	NR	NR	Favours control	
(S8) Ezie et al., 2022 [76]	Urine drug screens positive for opiates	39%^a^	36%^b^	Multiple logistic regression	> 0.05	Adjusted^g^ OR: 0.82 (0.34–1.98)	Favours intervention	Positive
	Urine drug screens positive for any non-prescribed substance other than cannabis	45%^a^	40%^b^	Multiple logistic regression	> 0.05	Adjusted^g^ OR: 0.61 (0.25–1.48)	Favours intervention	
(S15) Hoffman et al., 2022 [83]	Random monthly urine drug tests positive for opioids among clients in treatment for < 90 days	38% (SD 0.43)^a^	33% (SD 0.42)^b^	Wilcoxon rank sum test, Pearson's Chi-squared test	0.6	NR	Favours intervention	Positive
	Random monthly urine drug tests positive for opioids among clients in treatment for 90–180 days	19% (SD 0.34)^a^	33% (SD 0.43)^b^	Wilcoxon rank sum test, Pearson's Chi-squared test	0.041	NR	Favours control	
	Random monthly urine drug tests positive for opioids among clients in treatment for > 180 days	23% (SD 0.33)^a^	20% (SD 0.32)^b^	Wilcoxon rank sum test, Pearson's Chi-squared test	0.12	NR	Favours intervention	
	Expected change in random monthly urine drug test positivity per percentage point in take-home dosing above expected^h^	NR	NR	Linear regression	0.005	Slope: -0.12 (95% CI -0.21, -0.04)	Favours intervention	
(S22) Lintzeris et al., 2022 [90]	Any self-reported cannabis use	33%^a^	38%^n^	McNemar test	0.028	χ2: 4.817	Favours control	Positive
	Any self-reported benzodiazepine use	28%^a^	22%^n^	McNemar test	0.014	χ2: 6.017	Favours intervention	
	Any self-reported stimulant use	20%^a^	16%^n^	McNemar test	0.120	NR	Favours intervention	
	Any self-reported opioid use	30%^a^	24%^n^	McNemar test	0.033	χ2: 4.563	Favours intervention	
	Any self-reported injection drug use	29%^a^	22%^n^	McNemar test	0.077	NR	Favours intervention	
	Average days used among clients self-reporting cannabis use	Mean: 18.1 (SD 10.8) Median: 21^a^	Mean 18.0 (SD 11.0), Median 26^b^	Wilcoxon signed-rank test	0.020	Z: -2.331	Favours control	
	Average days used among clients self-reporting benzodiazepine use	Mean: 14.6 (SD 11.7) Median: 12^a^	Mean: 16.9 (SD 11.4) Median: 20^b^	Wilcoxon signed-rank test	NR	NR	Favours control	
	Average days used among clients self-reporting stimulant use	Mean: 6.5 (SD 8.2) Median: 3^a^	Mean: 5.9 (SD 7.4) Median: 3^b^	Wilcoxon signed-rank test	NR	NR	Favours intervention	
	Average days used among clients self-reporting opioid use	Mean: 12.2 (SD 10.7) Median: 8^a^	Mean: 7.9 (SD 9.1) Median: 4^b^	Wilcoxon signed-rank test	0.001	Z: -3.445	Favours intervention	
	Average days used among clients self-reporting injection drug use	Mean: 10.7 (SD 10.5) Median: 5^a^	Mean: 8.1 (SD 8.9) Median: 4^b^	Wilcoxon signed-rank test	0.010	Z: 2.577	Favours intervention	
	Percentage of clients with “statistically reliable” and “clinically relevant” increase in substance use (composite measure)^i^	43%^j^	(a) 40%^k^ (b) 17%^l^	Logistic regression	(a) p ≥ 0.05* (b) p < 0.05*	Adjusted OR: (a) 0.854 (0.39–1.87) (b) 0.273 (0.10–0.77)	Favours intervention	
(S25) Morin et al., 2021 [93]	Routine urine drug screens positive for fentanyl	Jan: 14% Feb: 13% Mar: 14%^a^	Apr: 12% May: 21% Jun: 26% Jul: 29% Aug: 29% Sep: 25%^b^	Fractional logistic regression	NR	OR: (a) Apr. vs. Jan: 0.9 (95% CI: 0.8–0.9) (b) May vs. Jan.: 1.7 (95% CI: 0.5–1.89) (c) Jun. vs. Jan.: NR (d) Jul. vs. Jan: NR (e) Aug. vs. Jan: 2.6 (95% CI: 2.3–2.9) (f) Sep vs. Jan: 2.2 (95% CI: 1.9–2.6)	Favours control^m^	Negative
	Routine urine drug screens positive for cocaine	Jan: 24% Feb: 24% Mar: 24%^a^	Apr: 23% May: 29% Jun: 28% Jul: 28% Aug: 26% Sep: 25%^b^	NR	NR	NR	Favours control^n^	
	Routine urine drug screens positive for methamphetamine	Jan: 18% Feb: 19% Mar: 20%^a^	Apr: 17% May: 23% Jun: 23% Jul: 18% Aug: 17% Sep: 19%^b^	NR	NR	NR	Favours control^n^	
	Routine urine drug screens positive for morphine	Jan: 13% Feb: 13% Mar: 13%^a^	Apr: 12% May: 15% Jun: 15% Jul: 15% Aug: 15% Sep: 15%^b^	NR	NR	NR	Favours control^n^	
	Routine urine drug screens positive for oxycodone	Jan: 6% Feb: 6% Mar: 6%^a^	Apr: 6% May: 7% Jun: 7% Jul: 6% Aug: 6% Sep: 6%^b^	NR	NR	NR	Favours control^n^	
(S30) Rosic et al., 2022 [98]	Percentage of opioid-positive urine drug screens	Mean: 7.5% (SD 17.2)^a^	Mean: 18.1% (SD 26.5)^b^	Paired t-test	p < 0.001	Risk difference: 10.56% (95% CI: 8.17–12.95)	Favours control	Mixed
	Percentage of clients with any opioid-positive urine drug screens	73.5%^a^	46.3%^b^	NR	NR	NR	Favours intervention	
(S37) Vicknasingam et al., 2021 [107]	Percentage of clients with urine toxicology tests positive for any illicit substance	Dec.: 23% Jan.: 23% Feb.: 18%^a^	Jun.: 24% Jul.: 19%^b^	NR	NR	NR	Favours control^n^	Negative

Where adjusted and unadjusted effect estimates were reported, we present adjusted estimates. In no case did this change the estimated direction of effect

*Acronyms*: *NR* not reported, *OR* odds ratio. Where bivariate and multivariate analyses were reported, we present the results of the multivariate analysis

^*^Not reported in the original study; inferred or calculated by authors

^a^Control group: OAT clients pre-pandemic

^b^Intervention group: OAT clients post-pandemic

^c^Control group: 2019 values from a fitted model that removed the main effect of year to “[capture] the effect of change in take-out schedule” (Bart et al., 2022, p. 3)

^d^Intervention group: 2020 values from a fitted model that removed the main effect of year

^e^Control group: All OAT clients

^f^Intervention group: OAT clients with additional take-home doses during pandemic

^g^Adjusted for years in treatment, age, substance use disorder diagnosis, psychiatric disorder diagnosis, and % reduction in visit frequency

^h^Analysis limited to clients with three months of pre-COVID-19 data and one month of post-COVID-19 data

^i^Defined as an increase of 4 or more days in the previous 28 days

^j^Control group: OAT clients with no take-home doses at follow up

^k^Intervention group (a): OAT clients with 1–5 take-home doses/week at follow up

^l^Intervention group (b): OAT clients with 6 + take-home doses/week at follow up

^m^Based on proportion of comparisons favouring control

^n^Based on mean control group value versus mean intervention group value

**Table 8:** Critical appraisal of quantitative studies reporting illicit substance use

No	Study	MMAT Section 3^a^ for quantitative non-randomized studies				
		1	**2**	3	4	5
		Are the participants representative of the target population?	Are measurements appropriate regarding both the outcome and intervention (or exposure)?	Are there complete outcome data?	Are the confounders accounted for in the design and analysis?^b^	During the study period, is the intervention administered (or exposure occurred) as intended?^b^
S4	Bart et al., 2022 [72]	Can't tell	Yes	Yes	Yes	No
S6	Corace et al., 2022 [74]	No	No	Yes	No	Yes
S8	Ezie et al., 2022 [76]	Yes	Can't tell	Yes	No	Can't tell
S15	Hoffman et al., 2022 [83]	No	Yes	Yes	Yes	Yes
S22	Lintzeris et al., 2022 [90]	Can't tell	Yes	Yes	Yes	No
S25	Morin et al., 2021 [93]	Yes	Yes	Can't tell	No	No
S30	Rosic et al., 2022 [98]	Yes	Yes	Yes	No	No
S37	Vicknasingam et al., 2021 [107]	Can't tell	Yes	Yes	No	Yes
# meeting quality criteria		3/8	6/8	7/8	3/8	2/8

^a^The MMAT (Mixed Methods Appraisal Tool) Qualitative Checklist is designed specifically for mixed methods systematic reviews (Hong et al., 2018 [57]). It consists of five sections specific to various study designs, each with five quality criteria. All quantitative studies included in this review, including quantitative components of mixed-methods studies, were appraised under *Sect. 3: Quantitative non-randomized studies*

^b^This review included studies in which the intervention of interest (relaxed restrictions on take-home doses) formed part of a broader intervention (e.g., pandemic-related changes to OAT treatment). To increase the relevancy of the quality assessments, we interpreted questions 4 and 5 relevant to the research question posed in this review

##### Negative direction

One time series study (S25) and two before-and-after studies (S4, S37) found an increase in the percentage of positive urine tests among OAT clients following pandemic-related treatment changes. One study (S4) used statistical modelling to examine whether urine test positivity was associated with number of take-home doses, but found no clear association.

##### Mixed direction

A cross-sectional study (S6) reported that clients receiving additional take-home doses during the pandemic were less likely to report increased or decreased opioid use since COVID-19. In a before-and-after study (S30), the total percentage of positive urine tests among OAT clients increased following the COVID-19 pandemic. However, the percentage of *clients* testing positive decreased.

##### Positive direction

Three before-and-after studies (S8, S15, S22) reported a decrease in the percentage of positive urine tests (S8, S15) or self-reported substance use (S22) following pandemic-related treatment changes. In one study (S15), a linear regression analysis limited to clients in treatment for at least three months before the pandemic found that the probability of a positive urine test decreased as take-home doses increased.

#### Fatal and non-fatal overdose

Seven studies reported measures of fatal and/or non-fatal overdose. The direction of effect was negative in one study (S6), mixed in four studies (S2, S13, S17, S26), and positive in two studies (S3, S8). See Table 9. Two studies were high-quality (S3, S13), one was moderate-quality (S2), and four were low-quality (S6, S8, S17, S26). Areas of concern included failure to account for confounding, unplanned co-interventions, and generalizability (see Table 10).

**Table 9:** Studies reporting measures of fatal and non-fatal overdose

Study	Measure	Control Group	Intervention Group	Statistical Test or Model	*p*-value	Estimate of Effect	Direction of Effect	Overall Effect Direction
(S2) Aldabergenov et al., 2022 [70]	Methadone-related deaths among people prescribed methadone	44 (95% CI: 37–50)^a^	55^b^	NR	≥ 0.05*	NR	Favours control	Mixed
	Buprenorphine-related deaths among people prescribed buprenorphine	2016: 1 2017: 1 2018: 1 2019: 1^c^	2020: 1^d^	NR	NR	NR	None	
(S3) Amram et al., 2021 [71]	Emergency department visits related to overdose	16^e^	15^f^	Chi-square, McNemar’s chi-square or Fisher’s exact test	1	NR	Favours intervention	Positive
	Odds of emergency department visit per one-dose difference in total take-home doses after regulatory changes	NR	NR	Generalized linear model with binary logistic function	1.73	Adjusted OR: 0.94 (0.86–1.03)	Favours intervention	
(S6) Corace et al., 2022 [74]	Self-reported opioid overdose(s) with or without emergency department visit	13%^g^	16%^h^	Chi square test	0.54	χ2: 0.37	Favours control	Negative
(S8) Ezie et al., 2022 [76]	“Overdose”, details not specified	2%^e^	0.7%^f^	Chi square test	> 0.05	NR	Favours intervention	Positive
(S13) Gomes et al., 2022 [81]	Non-fatal opioid overdoses^i^ among clients receiving daily methadone at baseline	9.5% per person-year^k^	6.9%/person-year^l^	Cox proportional-hazards model	< 0.05*	Weighted HR: 0.73 (95% CI: 0.56–0.96)	Favours intervention	Mixed
	Non-fatal opioid overdoses^i^ among clients receiving 5–6 take-home doses of methadone at baseline	1.8%/person-year^k^	1.4%/person-year^m^	Cox proportional-hazards model	≥ 0.05*	Weighted HR: 0.80 (95% CI: 0.50–1.28)	Favours intervention	
	Fatal opioid overdoses^j^ among clients receiving daily methadone at baseline	0.5% per person-year^k^	0.6%/person-year^l^	Cox proportional-hazards model	≥ 0.05*	Weighted HR: 1.26 (95% CI: 0.48–3.33)	Favours control	
	Fatal opioid overdoses^j^ among clients receiving 5–6 take-home doses of methadone at baseline	0.3%/person-year^k^	NR^m,n^	Cox proportional-hazards model	≥ 0.05*	Weighted HR: 0.48 (95% CI: 0.16–1.45)	Favours intervention	
	Non-fatal opioid overdoses^i^ among clients receiving daily buprenorphine/ naloxone at baseline	3.5%/person-year^k^	6.5%/person-year^m^	Cox proportional-hazards model	≥ 0.05*	Weighted HR: 1.86 (95% CI: 0.70–4.92)	Favours control	
	Non-fatal opioid overdoses^i^ among clients receiving 5–6 take-home doses of buprenorphine /naloxone at baseline	1.4%/person-year^k^	1.7%/person-year^m^	Cox proportional-hazards model	≥ 0.05*	Weighted HR: 1.23 (95% CI: 0.58–2.63)	Favours control	
(S17) Joseph et al., 2021 [85]	Non-fatal overdoses^o^	2^e^	6^f^	NR	NR	NR	Favours control	Mixed
	Fatal overdoses^o^	1^e^	0^f^	NR	NR	NR	Favours intervention	
(S26) Nguyen et al., 2021 [94]	Fatal overdoses^t^ among clients “established” in care without take-home doses at baseline	0.5%^q^	0.6%^r^	NR	≥ 0.05*	NR	Favours control	Mixed
	Fatal overdoses^p^ among clients “established” in care with take-home doses at baseline	4.1%^s^	0.8%^t^	NR	≥ 0.05*	NR	Favours intervention	

Where adjusted and unadjusted effect estimates were reported, we present adjusted estimates. Where weighted and unweighted effect estimates were reported, we present weighted estimates. In no case did this change the overall estimated direction of effect*Acronyms*: *HR *hazard ratio, *NR *not reported, *OR *odds ratio, *RR *relative risk

^*^Not reported in the original study; inferred or calculated by authors

^a^Control group: Projected deaths of methadone clients, Mar.-Jun. 2020

^b^Intervention group: Actual deaths of methadone clients, Mar.–Jun. 2020

^c^Control group: Buprenorphine clients, 2016–2019

^d^Intervention group: Buprenorphine clients, 2020

^e^Control group: OAT clients pre-pandemic

^f^Intervention group: OAT clients post-pandemic

^g^Control group: OAT clients without additional take-home doses during pandemic

^h^Intervention group: OAT clients with additional take-home doses during pandemic

^i^ ≥ 1 emergency department visit or inpatient hospitalization for opioid toxicity

^j^Coroner-confirmed fatal opioid overdoses

^k^Control group: OAT clients without additional take-home doses during pandemic

^l^Intervention group: OAT clients with additional take-home doses (any number) during pandemic

^m^Intervention group: Clients with additional take-home doses (at least a two-week supply) during pandemic

^n^Could not be modelled because of small numbers

^o^Overdoses reported to clinical personnel or documented in medical records

^p^Fatal overdoses ascertained from electronic health records; defined as death [over 10-month follow-up period] with any or multiple illicit substances (including opioids) listed as any of the potential causes of death

^q^Control group: Clients who never had take-home doses (neither before nor during pandemic)

^r^Intervention group: Clients newly started on take-home doses during pandemic

^s^Control group: Clients with no change or a decrease in take-home doses during pandemic

^t^Intervention group: Clients with additional take-home doses during pandemic

**Table 10:** Critical appraisal of quantitative studies reporting fatal and non-fatal overdose

No	Study	MMAT Section 3^a^ for quantitative non-randomized studies				
		1	2	3	4	5
		Are the participants representative of the target population?	Are measurements appropriate regarding both the outcome and intervention (or exposure)?	Are there complete outcome data?	Are the confounders accounted for in the design and analysis?^b^	During the study period, is the intervention administered (or exposure occurred) as intended?^b^
S2	Aldabergenov et al., 2022 [70]	Yes	Yes	Yes	No	No
S3	Amram et al., 2021 [71]	No	Yes	Yes	Yes	Yes
S6	Corace et al., 2022 [74]	No	No	Yes	No	Yes
S8	Ezie et al., 2022 [76]	Yes	Can't tell	Yes	No	Can't tell
S13	Gomes et al., 2022 [81]	Yes	Yes	Yes	Yes	Yes
S17	Joseph et al., 2021 [85]	Can't tell	No	No	No	No
S26	Nguyen et al., 2021 [94]	No	Yes	Yes	No	No
# meeting quality criteria		3/7	4/7	6/7	2/7	3/7

^a^The MMAT (Mixed Methods Appraisal Tool) Qualitative Checklist is designed specifically for mixed methods systematic reviews (Hong et al., 2018). It consists of five sections specific to various study designs, each with five quality criteria. All quantitative studies included in this review, including quantitative components of mixed-methods studies, were appraised under *Sect. 3: Quantitative non-randomized studies*

^b^This review included studies in which the intervention of interest (relaxed restrictions on take-home doses) formed part of a broader intervention (e.g., pandemic-related changes to OAT treatment). To increase the relevancy of the quality assessments, we interpreted questions 4 and 5 relevant to the research question posed in this review

##### Negative direction

A cross-sectional study (S6) found that self-reported opioid overdoses were higher for OAT clients who received extra take-home doses during the pandemic than for those who did not.

##### Mixed direction

A modelling study (S2) found that actual methadone-related deaths did not far exceed projected deaths among people prescribed methadone during England’s first COVID-19 lockdown, when most clients received two-week take-home doses. The count of buprenorphine-related deaths among people prescribed buprenorphine was unchanged compared with previous years. A retrospective, propensity-weighted cohort study found that increased take-home doses were associated with a lower risk of overdose among methadone clients and a higher risk among buprenorphine/naloxone clients (S13). In a commentary with data on overdoses reported to health care providers at opioid treatment programs in New York (S17), there was a higher count of non-fatal overdoses and a lower count of fatal overdoses after changes to take-home guidelines. A preprint with data on fatal overdoses among methadone clients (S26) reported that receiving additional take-home doses during the pandemic was associated with a higher rate of fatal overdose for clients without take-home doses at baseline. However, for clients who had take-home doses at baseline, those who received additional take-home doses during the pandemic had a lower rate of fatal overdose than those who did not.

##### Positive direction

One before-and-after study (S3) reported fewer overdose-related emergency department visits among methadone clients following changes to take-home guidelines. Statistical modelling showed that the odds of overdose decreased with each one-dose increase in take-home doses after controlling for age, gender, education, and employment. Another before-and-after study (S8) did not specify an outcome measure, but reported reduced overdoses among methadone clients following the relaxation of take-home guidelines.

#### Client health

Three studies reported on client health, which included measures of physical, emotional, and mental well-being and measures of infection and disability related to substance use. See Table 11. One study found a negative direction of effect (S8) and two reported a mixed direction of effect (S6, S2). One study was moderate-quality (S22) and two were low-quality (S6, S8). Sources of downgrading included generalizability, appropriateness of outcome measurements, failure to account for confounding, and unplanned co-interventions (see Table 12).

**Table 11:** Studies reporting measures of client health

Study	Measure	Control Group	Intervention Group	Statistical Test or Model	*p*-value	Estimate of Effect	Direction of Effect	Overall Effect Direction
(S6) Corace et al., 2022 [74]	Clients with self-reported visits to the emergency department “because of substance use”	9%^a^	9%^b^	Chi square test	0.98	χ2 = 0.00	No difference	Mixed
	Clients with self-reported admissions to the hospital “because of substance use”	7%^a^	12%^b^	Chi square test	0.15	χ2 = 2.05	Favours control	
(S8) Ezie et al., 2022 [76]	Incidence of new infectious disease (e.g., aspiration pneumonia, hepatitis, HIV, skin and soft tissue infections)	0%^c^	1.5%^d^	Chi square test	> 0.05	NR	Favours control	Negative
(S22) Lintzeris et al., 2022 [90]	Average scores on physical health scale (1 = poor, 10 = good)^e^	Mean: 6.6 (SD 1.8) Median: 7^c^	Mean 6.5 (SD 1.6) Median: 7^d^	Paired t-test	0.229	NR	Favours control	Mixed
	Average scores on psychological health scale (1 = poor, 10 = good)^e^	Mean 6.3 (SD 1.8) Median: 7^c^	Mean: 6.5 (SD 1.6) Median: 7^d^	Paired t-test	0.181	NR	Favours intervention	

Acronyms: *HR* hazard ratio, *NR* not reported, *OR* odds ratio, *RR* relative risk

^*^Not reported in the original study; inferred or calculated by authors

^a^Control group: OAT clients with at least one take-home dose during the pandemic, but without additional take-home doses

^b^Intervention group: OAT clients with additional take-home doses during the pandemic

^c^Control group: OAT clients pre-pandemic

^d^Intervention group: OAT clients post-pandemic

^e^Based on self-reported data collected through the Australian Treatment Outcome Profile

**Table 12:** Critical appraisal of quantitative studies reporting client health

No	Study	MMAT Section 3^a^ for quantitative non-randomized studies				
		1	2	3	4	5
		Are the participants representative of the target population?	Are measurements appropriate regarding both the outcome and intervention (or exposure)?	Are there complete outcome data?	Are the confounders accounted for in the design and analysis?^b^	During the study period, is the intervention administered (or exposure occurred) as intended?^b^
S6	Corace et al., 2022 [74]	No	No	Yes	No	Yes
S8	Ezie et al., 2022 [76]	Yes	Can't tell	Yes	No	Can't tell
S22	Lintzeris et al., 2022 [90]	Can't tell	Yes	Yes	Yes	No
# meeting quality criteria		1/3	1/3	3/3	1/3	1/3

^a^The MMAT (Mixed Methods Appraisal Tool) Qualitative Checklist is designed specifically for mixed methods systematic reviews (Hong et al., 2018). It consists of five sections specific to various study designs, each with five quality criteria. All quantitative studies included in this review, including quantitative components of mixed-methods studies, were appraised under *Sect. 3: Quantitative non-randomized studies*

^b^This review included studies in which the intervention of interest (relaxed restrictions on take-home doses) formed part of a broader intervention (e.g., pandemic-related changes to OAT treatment). To increase the relevancy of the quality assessments, we interpreted questions 4 and 5 relevant to the research question posed in this review

##### Negative direction

A study of methadone clients (S8) found that the incidence of infections associated with substance use was higher in the three months following the relaxation of restrictions on take-home doses than in the three months prior.

##### Mixed direction

A cross-sectional study (S6) found increased hospital admissions for substance use among OAT clients who received additional take-home doses during the pandemic, but no difference in emergency department visits for substance use. A before-and-after study (S22) using self-reported data reported a decrease in mean physical health scores and an increase in mean psychological health scores following pandemic-related changes to OAT.

#### Quality of life

Quality of life was reported in two studies. See Table 13. Direction of effect was negative in one low-quality study (S16) and positive in one moderate-quality study (S22). Both studies were downgraded for unplanned co-exposures (see Table 14).

**Table 13:** Studies reporting measures of quality of life

Study	Measure	Control Group	Intervention Group	Statistical Test or Model	*p*-value	Estimate of Effect	Direction of Effect	Overall Effect Direction
(S16) Javakhishvili et al., 2021 [84]	WHOQOL-BREF score, Physical Domain (0 = low, 100 = high)	Mean 58.95 (SD 14.82)^a^	Mean 57.24 (SD 16.22)^b^	NR	> 0.05	NR	Favours control	Negative
	WHOQOL-BREF score, Psychological Domain (0 = low, 100 = high)	Mean 59.11 (SD 10.12)^a^	Mean 57.04 (SD 10.73)^b^	NR	< 0.05	NR	Favours control	
	WHOQOL-BREF score, Social Domain (0 = low, 100 = high)	Mean 68.93 (SD 14.51)^a^	Mean 67.12 (SD 16.02)^b^	NR	> 0.05	NR	Favours control	
	WHOQOL-BREF score, Environmental Domain (0 = low, 100 = high)	Mean 53.51 (SD 11.9)^b^	Mean 52.5 (SD 12.39)^b^	NR	> 0.05	NR	Favours control	
(S22) Lintzeris et al., 2022 [90]	Australian Treatment Outcome Profile’s quality of life scale score (1 = low, 10 = high)	Mean 6.7 (SD 1.8)^c^	Mean 6.8 (SD 1.6)^d^	Paired t-test	0.157	NR	Favours intervention	Positive

^a^Control group: OAT clients who attended the OST site for medication every day during the pandemic

^b^Intervention group: OAT clients receiving take-home doses during pandemic

^c^Control group: OAT clients pre-pandemic

^d^Intervention group: OAT clients post-pandemic

**Table 14:** Critical appraisal of quantitative studies reporting quality of life

No	Study	MMAT Section 3^a^ for quantitative non-randomized studies				
		1	2	3	4	5
		Are the participants representative of the target population?	Are measurements appropriate regarding both the outcome and intervention (or exposure)?	Are there complete outcome data?	Are the confounders accounted for in the design and analysis?^b^	During the study period, is the intervention administered (or exposure occurred) as intended?^b^
S16	Javakhishvili et al., 2021 [84]	Yes	Yes	Can't tell	No	No
S22	Lintzeris et al., 2022 [90]	Can't tell	Yes	Yes	Yes	No
% meeting quality criteria		1/2	2/2	1/2	1/2	0/2

^a^The MMAT (Mixed Methods Appraisal Tool) Qualitative Checklist is designed specifically for mixed methods systematic reviews (Hong et al., 2018). It consists of five sections specific to various study designs, each with five quality criteria. All quantitative studies included in this review, including quantitative components of mixed-methods studies, were appraised under *Sect. 3: Quantitative non-randomized studies*

^b^This review included studies in which the intervention of interest (relaxed restrictions on take-home doses) formed part of a broader intervention (e.g., pandemic-related changes to OAT treatment). To increase the relevancy of the quality assessments, we interpreted questions 4 and 5 relevant to the research question posed in this review

##### Negative direction

A cross-sectional survey (S16) using the WHOQOL-BREF, a 26-item instrument for assessing quality of life, found that clients who received take-home doses had lower scores that those who continued to pick up their medication daily.

##### Positive direction

A before-and-after study (S22) found that OAT clients had higher scores on quality of life scales following pandemic-related changes to OAT.

#### Subgroup analysis

**Fig. 4:**
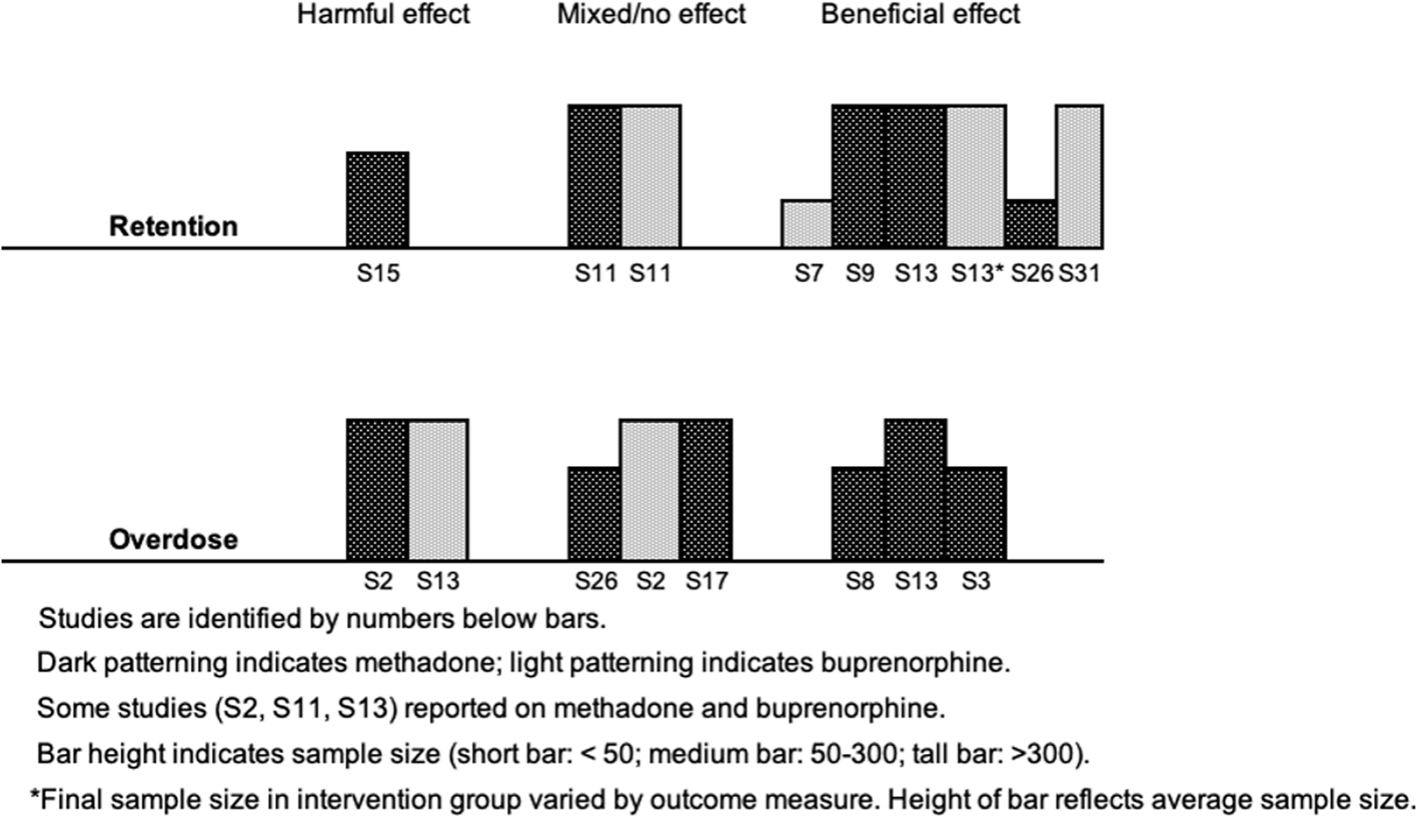
Subgroup analysis of retention and overdose by treatment type

**Fig. 5:**
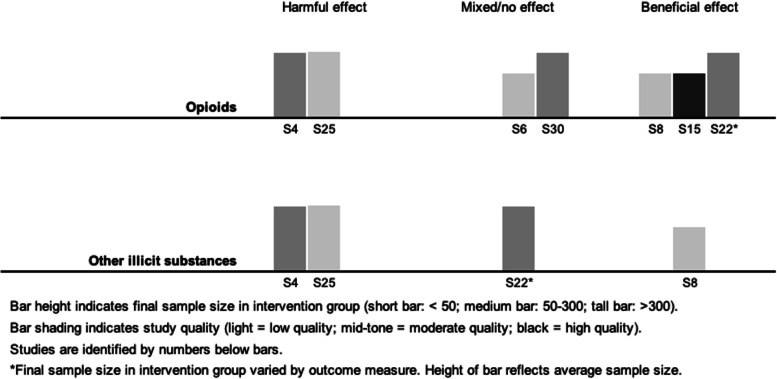
Exploratory subgroup analysis of illicit substance use

Subgroup analysis by treatment type (Fig. 4) showed no clear difference between methadone and buprenorphine in changes in retention and overdose. For all other outcomes, it was not possible to investigate differences between treatment types because of insufficient data (fewer than two buprenorphine studies). An unplanned subgroup analysis of illicit substance use by substance type (opioids versus other unregulated substances) was inconclusive, though the direction of effect was more often positive or mixed for use of unregulated opioids than for use of other unregulated substances (Fig. 5).

#### Sensitivity analysis

We explored the sensitivity of our findings to study quality by excluding low-quality studies (shown in light gray in Fig. 3). Visual inspection of harvest plots showed a decrease in the proportion of studies supporting a positive direction of effect for retention, although the overall trend was preserved. There were no notable changes in other outcome domains.

### Qualitative synthesis

We identified four analytical themes describing clients’ experiences with the relaxation of restrictions on take-home doses during COVID-19 (see Table 15). Clients’ quotes reflected a holistic view of treatment and indicated that access to take-home doses influenced self-perception, treatment experience, and mental health during the pandemic.

**Table 15:** Analytical themes resulting from qualitative synthesis

Theme #1: Feeling trusted to self-manage treatment	Clients felt trusted when they were provided with take-home doses. Take-home doses reassured them that they were doing well in treatment and increased pride, responsibility, and treatment autonomy.
Theme #2: Navigating environmental risks	Take-home doses allowed clients to reduce their exposure to triggers of illicit substance use and stigma. Reduced anxiety created space for experiences and environments that promoted client well-being.
Theme #3: Life/treatment balance	Take-home doses eliminated daily conflicts between treatment obligations and employment. With treatment consuming less of their time and energy, clients gave their attention to family and other rewarding activities.
Theme #4: Emotional and psychological impact of not receiving take-home doses	Clients who did not receive take-home doses during COVID-19 felt punished and exposed to unnecessary risk. Housing stability was a barrier to equitable treatment.

#### Theme #1: feeling trusted to self-manage treatment

Alongside meeting client’s physical needs, take-home doses increased client confidence. Offering this “bit of trust” (S33) made it possible for clients to reach a level of agency that previous medication policies did not allow. With more ownership of their medication, clients had the space and time to exercise their expertise in their own care and look after their needs (S23, S32–33, S35, S40). However, though some clients found it “very easy” (S20) to adapt to take-home doses and wanted to protect their right to keep them (S1, S15, S27), a few stated that they “had trouble with take-home doses” (S14) or were not “ready for it” (S27).

##### Reassurance and responsibility

Take-home doses were overwhelmingly seen as an indicator of trust (S1, S5, S15, S20, S33–34) between the clinician and the client. Take-home doses provided reassurance, signifying that clients “must be doing well” (S15) or were “on the right track” (S1, S15, S20) in their recovery.

*When you get your take-home doses it’s like you feel you are being trusted to take care of yourself, and do the right thing…it felt great…that I was on the right track in my recovery.* (Client in S20, p. 5)

In some cases, clients felt that take-home doses helped them move forward and gave them a sense of pride and personal achievement (S15, S20, S27).

*I feel that it's given me a sense of responsibility. I wasn't sure if I was ready to handle– but of course, I rose to the challenge. That makes me feel proud of myself.* (Client in S15, p. 4)

*I was much more physically stable because I wasn’t missing doses and also felt …it was sort of empowering as well, because it means they are trusting you to have the six takeaways, you felt more like a normal person, more like an adult, being trusted with some responsibility and that was quite empowering*. (Client in S5, p. 4)

##### Medication self-management

With more control over their medication, clients had the flexibility to adapt their dosing schedule to meet their individual needs (S5, S23, S32, S33, S35a, S40). Take-home doses functioned as a bridge to more autonomous care by enabling clients to take a more active role in managing their treatment (S5, S15, S19–20). Self-governance made it possible for clients to take their medication at a time that suited their needs, with some opting to take it later in the day (S15, S36a, S40) or preferring to split their dose (S12, S21, S23, S33). These aspects of medication ownership promoted better sleep (S5, S15, S36a) and helped clients navigate urges to use unregulated substances (S12, S15).

*That has been quite a … luxury to be able to have what I need at home and be able to dose at my convenience. I found that I like to take it at night, (it makes me feel better), but I can't do that if I'm going to the clinic every day.* (Client in S40, p. 1108)

*I was able to take my medication the way I was supposed to. I didn’t have to think of taking extra, I didn’t want to take extra.* (Client in S15, p. 4)

Though most clients associated take-home doses with positive experiences, a few felt “overwhelmed” (S20), self-identified as “addicts” (S1, S27), and were unsure of their ability to self-manage (S10, S20, S27).

*[…] For me [access to take-home doses] just wasn’t good at the time because I was still pretty new in my sobriety, you have to trust in yourself and everybody is different.* (Client in S20, p. 6)

*I basically told on myself and told [the clinic] that I was having trouble with the take-home doses, so they stopped giving them to me…I like it better because [going to the clinic] gets me up and ready for the day.* (Client in S14, p. 5)

A number of these clients had difficulty spacing out their doses and ran out of medication early (S1, S10, S16, S27). In some instances, they turned to unregulated opioids to ease the resulting withdrawal symptoms (S1, S27).

#### Theme #2: navigating environmental risks

Take-home doses promoted “less exposure” (S18) to imposed or perceived risks, including access to unregulated drugs and the threat of potential violence (S1, S10, S15, S18, S20–21, S34-35). When rigid protocols around medication access were lifted, clients who received take-home doses experienced reduced stigma and anxiety (S18, S34, S36, S38).

##### Wanting “less exposure”

Before the COVID-19 pandemic, clients were not given the option of distancing themselves from the “triggers on the street” (S1, S32) that some encountered during their clinic visits. Take-home doses acted as a protective “barrier” (S15), creating space between clients and the “old people” (S15, S21) and places that they preferred to “stay away” from (S20). Clients were able to manage their environments to protect their wellbeing and recovery by choosing to avoid situations where they were “reminded of [their] drug history all the time” (S21, p. 37).

*Cause when I would come here every day, I see people that I used with every day. And so when I am not seeing them every day I am getting a different type of habit. I am growing a different type of a habit outside of the clinic and so it's better for me that way I guess.* (Client in S15, p. 6)

Additionally, some clients with take-home doses stated that picking up their medication less frequently protected them from threats of theft or coercion (S23, S33).

##### Reduced stigma and anxiety

Compulsory clinic attendance for supervised dosing was seen as a “form of control” (S18) that created a constant fear of missing appointments and losing access to medication (S18, S34). With room to breathe, clients could create experiences and environments that were free from the stigma associated with receiving OAT (S18, S23, S34, S38).

*The good thing is I don’t have to keep going to the chemist which is a pain, a real pain [. . .] like they keep changing the pharmacist so you have to go through all the rigmarole of it being controlled and that, proving who you are and where you live and stuff*. (Client in S34)

Not all clients felt more protected from environmental risks, and some preferred to pick up their medication on a more frequent basis (S1, S28). In one example, a lack of safe and reliable housing increased the risk of medication theft (S1), while others had concerns around medication loss and spillage (S27, S36) or accidental consumption of the medication by others (S37).

#### Theme #3: life/treatment balance

Take-home doses reduced treatment burden and permitted clients to create space in their lives for employment, family, and rewarding daily activities. This facilitated a more “normal” life and made it easier for some clients to adhere to treatment (S5, S19, S24, S34). Reducing commutes to the clinic or pharmacy was particularly beneficial for clients balancing treatment with caregiving responsibilities (S15, S20, S38), physical disabilities (S15, S21, S34), mental health challenges (S19, S36a), or limited incomes (S15, S20–21, S34).

##### Employment

For working clients, daily supervised dosing created recurrent conflicts between treatment and employment (S1, S15, S19, S24, S29, S34, S38). Many contended with lengthy commutes (S21, S24), limited hours of service (S1, S24), and unpredictable wait times (S24, S34) to get their medication. Some clients reported that it was challenging to obtain or keep employment (S1, S29); others had missed doses (S19, S34) or been driven to give up treatment (S24, S34):

*(It) was a pain in the ass because the closest ([methadone] clinic) is in Bullhead. So they got to pick you up at five o’clock in the morning, drive you down there in the bus [. . .] you have to go all the way down there to see the doctor *[45 minutes]*. And there’s no guarantee you’re going to get your dose that day. And you have to sit there and wait and you make the bus wait. Well, after picking everybody up, you’re looking at like two hours, something like that. . ..That’s why I stopped going to them because I had to go to work. And there was no way I could make it all the way there to talk to the doctor and get everything set up, and then make it to work on time. There’s no possible way.* (Client in S24, p. 8)

Take-home doses made it possible for clients to meet their work commitments without compromising their treatment, and vice-versa.

*[. . .] I would miss days [before having take-home doses] because the window of time they’re open is limited and I work and have depression so I couldn’t get there every day. With take homes I’m far less likely to miss a dose and less likely to use.* (Client in S19, p. 5)

##### Family and rewarding daily activities

With greater control over their schedules, clients were “free” (S34) to give more attention to their families (S15, S20, S34, S36a) and to pursuing other rewarding activities (S1, S14–15, S20, S35a). These ranged from enjoying a leisurely morning coffee (S14) to going to the gym (S35a) and spending time outdoors:

*[Having more take-home doses] gives me a little break. [I can do] other things, like going to the river. I went and floated this weekend, and just hanging out with dad and barbecuing and doing yard work and stuff like that.* (Client in S20, p. 5)

##### Benefits of daily supervised dosing

A smaller number of clients missed the daily routine of supervised dosing (S14, S18, S21, S28). For these individuals, picking up their dose each day gave them “a reason to get out of the house” (S28, p. 12) and ongoing access to healthcare and social supports (S14, S21, S27):

*When you're on the clinic, you go every single day, which means you got to get up and leave the house [. . .] In a way, [getting take-home doses] helped me, but then in a way it hurts too because I started that feeling again of not leaving the house…I think I probably shouldn't have got any take-home doses and just continued going daily, and seeing the nurses and the counselors that were there*. (Client in S14, p. 4).

#### Theme #4: emotional and psychological impact of not receiving take-home doses

Though some clients received additional take-home doses during the pandemic, others were required to continue with daily supervised dosing (S5, S40). Although their treatment was unchanged on the surface, the relaxation of restrictions on take-home doses had a profound emotional and psychological impact on many of these clients.

##### Anger and frustration with differential treatment

Clients who continued to pick up their medication daily were acutely aware of the risk of COVID-19 infection during these visits (S14, S19, S38). Being forced to run “that germ gauntlet” (S19, p. 4) spurred anger and frustration, particularly given that other aspects of society had been radically overhauled to protect the general public:

*I still had to get up and go [pick up methadone] every day. They weren't running trains. They weren't running the buses…I'm five miles away from [the] inner city. And here I am having to fucking ride the bike down the highway…We couldn't do anything [during the pandemic], but it's okay to send the drug addicts out. The homeless guys out so that they can go get their food stamps and fucking methadone.* (Client in S14, p. 5)

Clients whose take-home doses were revoked after the early phases of the pandemic also expressed dissatisfaction:

*I don’t like [going from one month to 2 weeks] at all but, honestly, you don’t rattle the cage too much…I feel kind of put upon in a way because…I shouldn’t be in there with all the people. I am staying away from the grocery stores and everything but my methadone—of course. Anyway, I am not happy, but I’m not mad either. Just disappointed …* (Client in S20, p. 5)

##### Supervised dosing as punitive

The feeling that supervised dosing was “punishment”, either for substance use generally or for the behaviour of a minority of people using substances, was pervasive among clients (S21, S24, S35a, S40):

*[. . .] heroin addicts are, I believe, hated by society so there's a whole idea that you have to suffer … or be controlled. Otherwise, you're gonna do yourself some harm.* (Client in S40, p. 1108)

While some clients viewed daily supervised dosing as appropriate in certain cases (S1, S15, S34), particularly for those who were just beginning treatment [34], a common sentiment was that restrictions on take-home doses were crudely applied and needed to accommodate greater consideration of individual circumstances (S19–20, S24, S34).

In contrast, a few individuals felt that restrictions on take-home doses encouraged clients to be “dedicated” (S15) to their treatment adherence or abstinence (S15, S34), with one client explaining that having their take-home doses rescinded “gave me time to really acknowledge where I really messed up” (S1, p. 5).

##### Compounding inequities

Clients who remained on take-home doses found clinics busier than usual (S18, S38), perhaps because of shorter hours of operation, social distancing measures, and reduced transit schedules (S14). Social distancing meant that some clients had to line up outside, where they felt conspicuous and exposed to judgment:

*Since the whole virus thing they’ve been like it has been like really packed, so to have to wait on line outside a lot it’s embarrassing and I’m feeling things oh, look at them the drug addicts.* (Client in S14, p. 1148)

Housing stability influenced access to take-home doses (S14, S35b). For clients with stable housing, the pandemic brought take-home doses into the realm of possibility; for those without, it cast their ineligibility into sharp relief:

*It's also been very difficult trying to stay clear of the virus…I didn't qualify for take-home doses. I don't have a home to take [methadone] to. I didn't qualify for a lockbox full of meds that I could give to anybody that was in a position of being able to watch me. Because nobody's in that position over me, I'm homeless [. . .]* (Client in S14, p. 5)

In this way, the liberalization of take-home doses increased treatment inequity for clients with unstable housing.

#### Sensitivity analysis

The majority of studies contributing to each qualitative theme were appraised as high-quality (see Tables 16, 17, 18 and 19). Excluding low- and moderate-quality studies from the synthesis did not change the findings appreciably.

**Table 16:** Critical appraisal of qualitative studies supporting Theme #1: feeling trusted to self-manage treatment

No	Study	MMAT Sect. 1^a^ for qualitative studies				
		1	2	3	4	5
		Is the qualitative approach appropriate to answer the research question?	Are the qualitative data collection methods adequate to address the research question?	Are the findings adequately derived from the data?	Is the interpretation of results sufficiently substantiated by data?	Is there coherence between qualitative data sources, collection, analysis and interpretation?
S1	Abidogun et al., 2023 [69]	Yes	Yes	Yes	Yes	Yes
S5	Conway et al., 2023 [73]	Yes	Yes	Yes	Yes	Yes
S10	Gage et al., 2022 [78]	Yes	Yes	Yes	Yes	Yes
S12	Gittins et al., 2022 [80]	Yes	No	Can't tell	Yes	Yes
S14	Harris et al., 2022 [82]	Yes	Yes	Yes	Yes	Yes
S15	Hoffman et al., 2022 [83]	Yes	Yes	Yes	Yes	Yes
S16	Javakhishvili et al., 2021 [84]	Yes	Yes	Can't tell	Yes	Yes
S19	Krawczyk et al., 2021 [87]	Yes	Yes	Yes	Yes	Yes
S20	Levander et al., 2021 [88]	Yes	Yes	Yes	Yes	Yes
S21	Liddell et al., 2021 [89]	Yes	Yes	Can't tell	Yes	Yes
S23	May et al., 2022 [91]	Yes	Yes	Yes	Yes	Yes
S27	Nobles et al., 2021 [95]	Yes	No	Yes	Yes	Yes
S32	Russell et al., 2021 [100]	Yes	Yes	Yes	Yes	Yes
S33	Schofield et al., 2022 [101]	Yes	Yes	Yes	Yes	Yes
S34	Scott et al., 2023 [102]	Yes	Yes	Yes	Yes	Yes
S35	Suen et al., 2022/Wyatt et al., 2022 [104]	Yes	Yes	Yes	Yes	Yes
S36	University of Bath et al., 2020, 2021 [106]	Can't tell	Yes	Can't tell	Can't tell	Can't tell
S40	Zhen-Duan et al., 2022 [110]	Yes	Yes	Yes	Yes	Yes
# meeting quality criterion		17/18	16/18	14/18	17/18	17/18

^a^The MMAT (Mixed Methods Appraisal Tool) Qualitative Checklist is designed specifically for mixed methods systematic reviews (Hong et al., 2018). It consists of five sections specific to various study designs, each with five quality criteria. All qualitative studies included in this review including qualitative components of mixed-methods studies, were appraised under *Sect. 1: Qualitative studies*

**Table 17:** Critical appraisal of qualitative studies supporting Theme #2: navigating environmental risks

No	Study	MMAT Sect. 1^a^ for qualitative studies				
		1	2	3	4	5
		Is the qualitative approach appropriate to answer the research question?	Are the qualitative data collection methods adequate to address the research question?	Are the findings adequately derived from the data?	Is the interpretation of results sufficiently substantiated by data?	Is there coherence between qualitative data sources, collection, analysis and interpretation?
S1	Abidogun et al., 2023 [69]	Yes	Yes	Yes	Yes	Yes
S10	Gage et al., 2022 [78]	Yes	Yes	Yes	Yes	Yes
S15	Hoffman et al., 2022 [83]	Yes	Yes	Yes	Yes	Yes
S18	Kesten et al., 2021 [86]	Yes	Yes	Yes	Yes	Yes
S20	Levander et al., 2021 [88]	Yes	Yes	Yes	Yes	Yes
S21	Liddell et al., 2021 [89]	Yes	Yes	Can't tell	Yes	Yes
S23	May et al., 2022 [91]	Yes	Yes	Yes	Yes	Yes
S27	Nobles et al., 2021 [95]	Yes	No	Yes	Yes	Yes
S28	Parkes et al., 2021 [96]	Yes	Yes	Yes	Yes	Yes
S32	Russell et al., 2021 [100]	Yes	Yes	Yes	Yes	Yes
S33	Schofield et al., 2022 [101]	Yes	Yes	Yes	Yes	Yes
S34	Scott et al., 2023 [102]	Yes	Yes	Yes	Yes	Yes
S36	University of Bath et al., 2020, 2021 [106]	Can't tell	Yes	Can't tell	Can't tell	Can't tell
S37	Vicknasingam et al., 2021 [107]	Can't tell	Yes	Can't tell	No	No
S38	Walters et al., 2022 [108]	Yes	Yes	Yes	Yes	Yes
# meeting quality criterion		13/15	14/15	12/15	13/15	13/15

^a^The MMAT (Mixed Methods Appraisal Tool) Qualitative Checklist is designed specifically for mixed methods systematic reviews (Hong et al., 2018). It consists of five sections specific to various study designs, each with five quality criteria. All qualitative studies included in this review including qualitative components of mixed-methods studies, were appraised under *Sect. 1: Qualitative studies*

**Table 18:** Critical appraisal of qualitative studies supporting Theme #3: life/treatment balance

No	Study	MMAT Sect. 1^a^ for qualitative studies				
		1	2	3	4	5
		Is the qualitative approach appropriate to answer the research question?	Are the qualitative data collection methods adequate to address the research question?	Are the findings adequately derived from the data?	Is the interpretation of results sufficiently substantiated by data?	Is there coherence between qualitative data sources, collection, analysis and interpretation?
S1	Abidogun et al., 2023 [69]	Yes	Yes	Yes	Yes	Yes
S14	Harris et al., 2022 [82]	Yes	Yes	Yes	Yes	Yes
S15	Hoffman et al., 2022 [83]	Yes	Yes	Yes	Yes	Yes
S18	Kesten et al., 2021 [86]	Yes	Yes	Yes	Yes	Yes
S19	Krawczyk et al., 2021 [87]	Yes	Yes	Yes	Yes	Yes
S20	Levander et al., 2021 [88]	Yes	Yes	Yes	Yes	Yes
S21	Liddell et al., 2021 [89]	Yes	Yes	Can't tell	Yes	Yes
S24	Meyerson et al., 2022 [92]	Yes	Yes	Can't tell	Can't tell	Can't tell
S27	Nobles et al., 2021 [95]	Yes	No	Yes	Yes	Yes
S28	Parkes et al., 2021 [96]	Yes	Yes	Yes	Yes	Yes
S29	Pilarinos et al., 2022 [97]	Yes	Yes	Yes	Yes	Yes
S34	Scott et al., 2023 [102]	Yes	Yes	Yes	Yes	Yes
S35	Suen et al., 2022/Wyatt et al., 2022 [104]	Yes	Yes	Yes	Yes	Yes
S36	University of Bath et al., 2020, 2021 [105]	Can't tell	Yes	Can't tell	Can't tell	Can't tell
S38	Walters et al., 2022 [108]	Yes	Yes	Yes	Yes	Yes
S39	Watson et al., 2022 [109]	Yes	Yes	Yes	Yes	Yes
# meeting quality criterion		15/16	15/16	13/16	14/16	14/16

^a^The MMAT (Mixed Methods Appraisal Tool) Qualitative Checklist is designed specifically for mixed methods systematic reviews (Hong et al., 2018). It consists of five sections specific to various study designs, each with five quality criteria. All qualitative studies included in this review including qualitative components of mixed-methods studies, were appraised under *Sect. 1: Qualitative studies*

**Table 19:** Critical appraisal of qualitative studies supporting Theme #4: emotional and psychological impact of not receiving take-home doses

No	Study	MMAT Sect. 1^a^ for qualitative studies				
		1	2	3	4	5
		Is the qualitative approach appropriate to answer the research question?	Are the qualitative data collection methods adequate to address the research question?	Are the findings adequately derived from the data?	Is the interpretation of results sufficiently substantiated by data?	Is there coherence between qualitative data sources, collection, analysis and interpretation?
S1	Abidogun et al., 2023 [69]	Yes	Yes	Yes	Yes	Yes
S14	Harris et al., 2022 [82]	Yes	Yes	Yes	Yes	Yes
S15	Hoffman et al., 2022 [83]	Yes	Yes	Yes	Yes	Yes
S18	Kesten et al., 2021 [86]	Yes	Yes	Yes	Yes	Yes
S19	Krawczyk et al., 2021 [87]	Yes	Yes	Yes	Yes	Yes
S20	Levander et al., 2021 [88]	Yes	Yes	Yes	Yes	Yes
S21	Liddell et al., 2021 [89]	Yes	Yes	Can't tell	Yes	Yes
S24	Meyerson et al., 2022 [92]	Yes	Yes	Can't tell	Can't tell	Can't tell
S34	Scott et al., 2023 [102]	Yes	Yes	Yes	Yes	Yes
S35	Suen et al., 2022/Wyatt et al., 2022 [104]	Yes	Yes	Yes	Yes	Yes
S38	Walters et al., 2022 [108]	Yes	Yes	Yes	Yes	Yes
S40	Zhen-Duan et al., 2022 [110]	Yes	Yes	Yes	Yes	Yes
# meeting quality criteria		12/12	12/12	10/12	11/12	11/12

^a^The MMAT (Mixed Methods Appraisal Tool) Qualitative Checklist is designed specifically for mixed methods systematic reviews (Hong et al., 2018). It consists of five sections specific to various study designs, each with five quality criteria. All qualitative studies included in this review including qualitative components of mixed-methods studies, were appraised under *Sect. 1: Qualitative studies*

### Integrated analysis

**Fig. 6:**
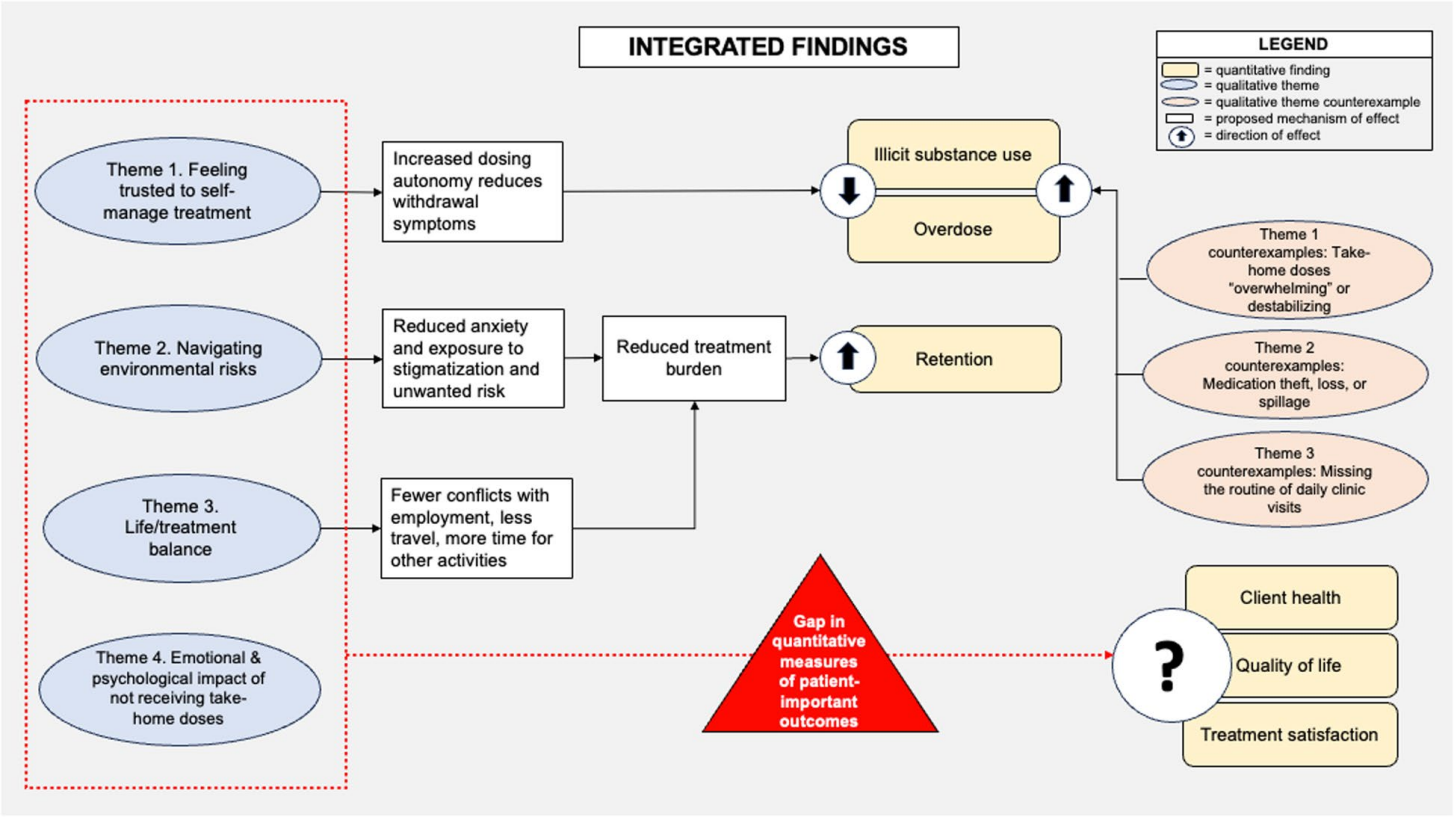
Visual representation of integrated findings

We juxtaposed the quantitative and qualitative syntheses and found that the qualitative findings provided a plausible mechanism for the increased retention observed in the quantitative studies. We did not observe any evidence of an association between take-home doses, illicit substance use, and overdose risk in the quantitative synthesis. However, the qualitative findings suggested that this apparent lack of association may conceal individual variation in the impact of take-home doses. We identified a critical gap in the quantitative literature on quality of life, client health, and treatment satisfaction. See Fig. 6 for a visual representation of the integrated findings.

#### Reduced treatment burden observed in qualitative evidence may explain increased retention

The qualitative evidence suggests that reduced treatment burden may account for the increased retention observed in the quantitative synthesis. Definitions of treatment burden vary; however, it has been characterized as a multidimensional concept that includes the “physical, financial, temporal, and psychosocial” demands that treatment imposes on patients (Sav et al., 2013). Tran et al. (2014) take a similarly holistic view, describing treatment burden as “the ‘work of being patient and its effect on the quality of life [...] the challenges associated with everything patients have to do to take care of themselves” (p. 2).

In the qualitative synthesis, the burden of treatment included the costs of travelling to the clinic and the opportunity costs of losing or being unable to obtain employment because of conflicts with daily supervised dosing requirements. Several clients explicitly linked employment to missed doses or treatment discontinuation. Others discussed the physical and time burden of treatment; physical disabilities, mental health challenges, and caregiving responsibilities were described as challenges to frequent clinic attendance.

In addition, daily supervised dosing generated significant psychosocial burden. Inflexible treatment conditions forced clients to repeatedly subject themselves to environments where they felt mistrusted, stigmatized, and anxious about encountering substance use triggers. Take-home doses, in enabling clients to avoid negative experiences that reinforced “addict” identities, may have made them more likely to stay in treatment.

#### Individual variation in illicit substance use and overdose risk

The quantitative synthesis showed no evidence of an association between take-home doses and illicit substance use or overdose. It is possible that this finding conceals differences between subgroups, as the qualitative analysis showed individual variation in the relationship between take-home doses, illicit substance use, and overdose risk.

Some clients stated that take-home doses reduced their exposure to people and environments associated with use of unregulated substances. Others noted that take-home doses meant fewer missed doses and allowed them to administer their medication in a way that increased its perceived efficacy: for instance, through splitting their dose or taking it a preferred time of day. A few of these clients reported reduced withdrawal symptoms, allowing them to reduce their use of unregulated substances and, by extension, risk of overdose.

However, though most clients described positive experiences with take-home doses, a small number of individuals preferred the structure and accountability of daily dosing and had difficulty regulating their use of medication when given a multi-day supply. Two studies described instances of clients turning to the unregulated drug market, increasing their overdose risk, after consuming their medication before their next scheduled pick-up date.

Taken together, the qualitative and quantitative syntheses suggest that take-home doses may have decreased illicit substance use for some clients while increasing use within the smaller group of clients who experienced take-home doses as destabilizing. One of the primary studies in this review (S30) supports this hypothesis. The authors of this study reported that the percentage of urine tests positive for opioids in a cohort of OAT clients increased by an average of 10.6% during COVID-19, but that the percentage of clients abstinent from opioid use (defined as zero positive urine tests) increased from 26.5% to 53.7%, despite no significant change in the median number of urine tests per month.

#### Key facets of client experience not captured by quantitative studies

We identified no quantitative studies reporting on treatment satisfaction and very few studies reporting on client health or quality of life. The findings of the qualitative synthesis suggest that this is a significant gap. In describing the impact that take-home doses had on their lives, most clients focused on how take-home doses affected their perceptions of themselves, their experiences of treatment, and their mental health. Relatively few focused on the impact of changes on their use of illicit substances or risk of overdose, which, together with retention, were the most frequently reported outcomes in the quantitative studies.

## Discussion

In this review, the relaxation of restrictions on take-home doses during the COVID-19 pandemic was associated with improved client experience and increased retention in OAT. We found no evidence that offering take-home doses to previously ineligible clients altered rates of illicit substance use or overdose in this population. We note that the risk of overdose in the community (i.e., from diverted medication) is also an important consideration. However, the scope of the present review was limited to the impact of take-home doses on individuals in treatment. Our findings align with the results of a recent policy review of the evidence on pandemic-related regulatory changes to methadone treatment in the United States [49]. Previous systematic reviews of supervised versus unsupervised dosing did not identify any studies of overdose and found no evidence of a difference in retention or illicit substance use [22, 23]. In both reviews, however, the authors concluded that the size and quality of the evidence base prevented them from drawing conclusions [22, 23].

### Treatment burden and retention in treatment

Our qualitative findings suggested that reduced treatment burden may explain the association between take-home doses and increased retention. There is growing recognition of the impact of treatment burden on people managing chronic conditions [111–113]. Studies show a significant association between treatment burden and medication adherence; as burden increases, adherence decreases [113, 114]. In OAT, lower adherence may translate into lower retention because missed doses reduce medication effectiveness. In addition, the substance use that may result from missed doses can result in treatment dismissal in some OAT programs [18].

Validated instruments for measuring treatment burden are a relatively recent development and have rarely been used in OAT [115]. However, research supports an association between various dimensions of treatment burden and retention in OAT. For instance, retention decreases when the time burden of treatment is increased, as when treatment includes mandatory counselling [116] or when clients travel more than 30 min to reach their clinic [117].

The difficulty of balancing treatment and employment is widely recognized as a barrier to retention [36, 118, 119]. In addition to anecdotal evidence of clients leaving treatment because of work conflicts [117, 120–123], a recent cohort study found that employment was a significant predictor of “sub-optimal care trajectories” in OAT [124]. Stigma is a compounding factor, as reluctance to disclose OAT may discourage clients from seeking accommodations from their employers [125].

Commentators have responded to the growing body of research on treatment burden with calls for “minimally disruptive medicine” that recognizes the impact of treatment demands, such as supervised dosing requirements, on clients’ lives [17, 126]. The findings of the present review suggests a need for further research using validated instruments to measure treatment burden in OAT.

### Optimizing the benefits of take-home doses

In the integrated analysis, we concluded that an apparent lack of association between take-home doses, illicit substance use, and overdose may obscure differences in the impact of take-home doses on individual clients. Previous qualitative studies also show divergence in client experiences, with some clients preferring supervision [127] or stating that a short period of supervision is helpful upon treatment entry [29, 128].

In the present review, as in previous studies [29, 129], clients had insight into their ability to manage take-home doses. These findings suggest that the benefits of take-home doses can be optimized by treating clients as active participants in care planning. Retaining flexibilities around take-home doses in the post-COVID-19 era would allow providers and clients to evaluate the merits of take-home doses relative to individual treatment needs and preferences. Research supports the value of client engagement in improving experiences of treatment [130–132], enhancing therapeutic relationships [131, 133], and determining effective dosages in OAT [134].

Based on the qualitative synthesis, factors that warrant discussion between providers and clients include the client’s level of comfort with a higher degree of self-management, the benefits and disadvantages of decreased clinic attendance, and the impact of supervised dosing on the client’s life/treatment balance. These discussions may occur in conjunction with consideration of other factors affecting individual risk, such as ongoing use of unregulated opioids. Findings also suggest that the option to return to supervised dosing if desired should be available to clients who request take-home doses.

### Split dosing and medication effectiveness

In the qualitative synthesis, some OAT clients identified their ability to time their medication or split their dose as an advantage of take-home doses. Methadone is typically offered to OAT clients once a day because its average half-life approximates 24 h [135]. However, medication interactions and wide variations in individual metabolism mean that some people on this regimen will have breakthrough withdrawal symptoms that cannot be resolved through a simple increase in dose [135]. In a recent pharmacokinetic study, serum testing showed that 8.5% of the sample were ultra-rapid methadone metabolizers who would benefit from split dosing [136].

Increased access to split dosing may also benefit the 55–61% of methadone clients who report chronic pain [137]. Management of pain in OAT clients is complicated by uncertainties around best practices [138], stigma and distrust from health care providers [139], and the complex relationship between pain and opioid use [140]. Though methadone is not a first line treatment for pain in the general population, a recent systematic review suggests that a divided dose of methadone may be preferable to other opioid analgesics for some methadone clients with chronic pain [138]. However, research in this area consists primarily of case series and case reports [138]. For OAT clients using methadone for analgesia, multiple daily doses are necessary because methadone does not provide pain relief for as long as it suppresses withdrawal. Clients who use unregulated substances to alleviate chronic pain are unlikely to get the same benefit from once-daily methadone.

Relaxed restrictions on take-home doses, in making split dosing more accessible to clients, may increase medication effectiveness for rapid metabolizers while supporting treatment regimens that combine opioid maintenance with methadone for analgesia.

### Patient-important outcomes

Of the quantitative outcomes included in this review, the most frequently reported were retention, substance use, and overdose. Given that clients in the qualitative synthesis focused primarily on the impact of take-home doses on their psychological state and life/treatment balance, relatively few quantitative studies reported on client health, quality of life, or treatment satisfaction. This is consistent with previous research demonstrating that common measures of effectiveness in OAT do not necessarily reflect the outcomes valued by clients [141–145].

Though reducing use of unregulated substances is a common treatment goal [142], many OAT clients also seek improved psychological wellbeing, improved relationships, improved role functioning, and decreased stigma and shame [132, 143, 146]. Reed et al. (2023) found that clients asked to rate the importance of predefined recovery goals considered “having a sense of self-worth” as important as “not using opioids” [146] while Treloar et al. (2007) reported that clients valued take-home doses for making them feel trusted [147]. Numerous studies have found that clients also value “feeling normal” or “living a normal life” [142, 143, 147, 148] – sentiments echoed by clients in the present review.

Recent studies have highlighted the limitations of traditional outcome measures and established the need for greater consideration of outcomes important to clients [142, 144]. In the qualitative studies included in the present review, clients valued the take-home doses that they received during the pandemic in part for their impact on psychological well-being. Substance use disorders are closely intertwined with anxiety, mood disorders, and other mental health challenges [149], and there is clear value in treatment delivery models and outcome measures that reflect the importance of meeting clients’ mental health needs during OAT. To our knowledge, there is not yet a widely accepted set of patient-important outcomes for use in recovery from substance use disorder, although at least one such instrument has been developed [150]. Involving people with lived and living experience of substance use in the development of patient-important outcome measures is essential to ensuring that they are relevant and meaningful to clients [150–153].

### Strengths and limitations

The relaxation of restrictions on take-home doses occurred in conjunction with other changes to program delivery, such as increased use of telehealth and reduced frequency of urine testing. In the case of buprenorphine, which was subject to fewer restrictions than methadone pre-pandemic, the impact of these changes may have exceeded the impact of the relaxation of restrictions on take-home doses. However, few of the studies identified in this review focused exclusively or primarily on buprenorphine. The pandemic itself was associated with social upheaval, changes to the unregulated drug supply, and disruptions to harm reduction services [154, 155]. It was not possible to control for these confounders in the quantitative synthesis. However, we were able to mitigate this limitation by using a mixed methods approach that allowed us to triangulate the quantitative findings with qualitative data. In this review, the qualitative findings were consistent with an association between take-home doses and retention and suggested treatment burden as a plausible explanation. Nevertheless, the association that we observed between take-home doses and retention should be interpreted with caution, particularly given that a sensitivity analysis excluding low-quality studies weakened the evidence supporting a positive direction of effect for retention. We also note that the impact of take-home doses may have been influenced by factors that we could not fully account for in this review, such as the level of pre-pandemic restrictions, the flexibilities provided by guidelines issued during the pandemic, and the extent to which flexibilities were implemented. These are known to have varied substantially [47].

We synthesized the quantitative findings using vote counting based on direction of effect. This method is preferable to simple narrative synthesis in that it reduces bias in the presentation and interpretation of findings [59]. It also has limitations. First, it provides no information about magnitude of effect [59]. Though we found evidence of a positive association between take-home doses and retention, we are unable to conclude whether the size of this increase would be considered meaningful in a clinical setting.

Second, vote counting based on direction of effect is less powerful than other methods of synthesis [59]. Compounding this limitation is the fact that a number of the quantitative studies used a before-and-after design that did not distinguish between clients who benefited from relaxed restrictions and those who remained on supervised dosing during the pandemic. This may have masked any associations between take-home doses and program effectiveness. Our finding of no association between take-home doses, illicit substance use, and overdose cannot be considered conclusive, particularly as the qualitative synthesis indicated that take-home doses were widely perceived as facilitating recovery.

### Deviations from protocol

This review deviated from our protocol in that we did not contact subject matter experts to solicit unpublished manuscripts or re-run all searches prior to the final analysis. However, several of the databases that we searched included preprints (e.g., Ovid MEDLINE ALL; Embase) and we conducted an additional round of forward citation chaining on Mar. 31, 2022, to capture articles published after the initiation of this review. We engaged with OAT clients by discussing our preliminary findings with seven community members with lived experience of OAT rather than through the town hall approach specified in our original research protocol.

## Conclusions

**Fig. 7:**
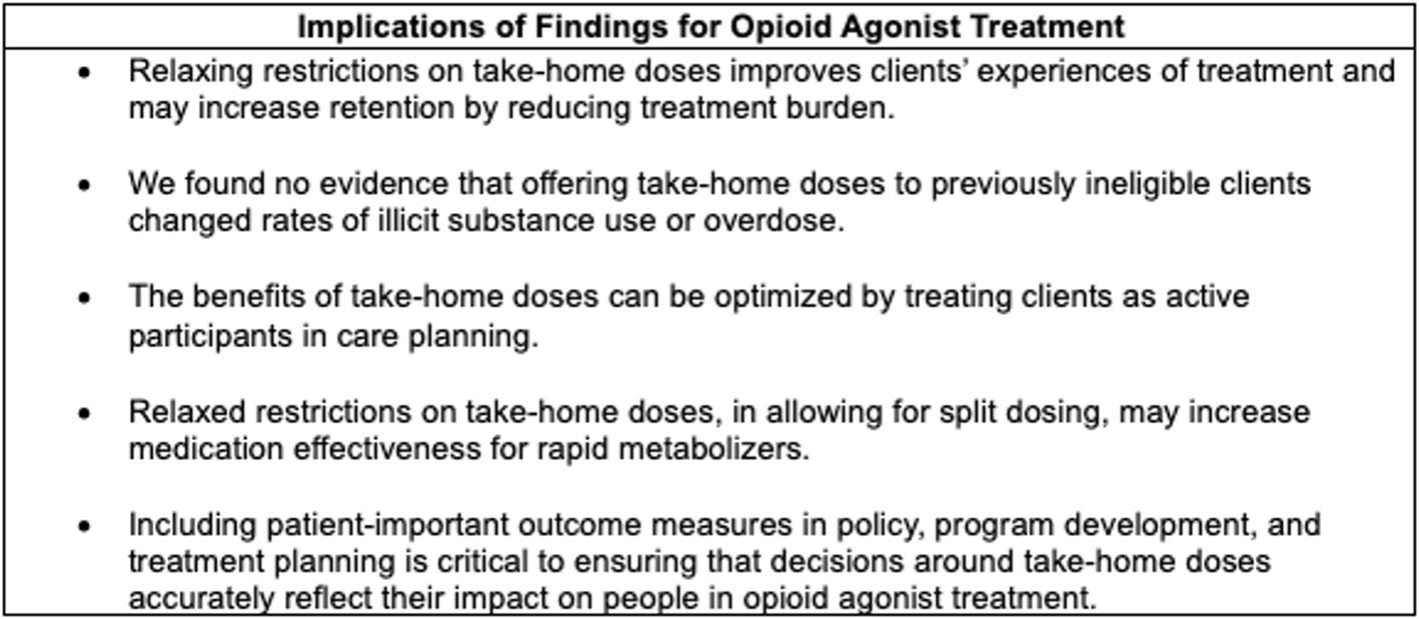
Implications of findings for opioid agonist treatment

In this mixed methods systematic review, we found that the relaxation of restrictions on take-home doses during the COVID-19 pandemic was associated with increased retention in OAT. See Fig. 7 for a summary of the implications of our findings for opioid agonist treatment. Qualitative evidence suggested that changes in retention may be attributable to reduced treatment burden. We found no evidence of an association between take-home doses and illicit substance use or overdose, despite the expansion of take-home doses to individuals who were ineligible to receive them prior to the pandemic.

Previous qualitative studies have demonstrated that daily supervised dosing is burdensome, stigmatizing, and viewed with disfavour by many clients [33, 34, 156, 157]. This review builds on that body of research by illuminating the ways in which more liberal provision of take-home doses altered clients’ experiences of treatment during the COVID-19 pandemic. Though some clients reported challenges with managing their medication, the dominant narrative was one of appreciation, reduced anxiety, and a renewed sense of agency and identity.

Crucially, these benefits are not captured by traditional measures of effectiveness in OAT. This suggests that pre-pandemic policies on take-home doses severely underestimate their value to clients. Including patient-important outcome measures in policy, program development, and treatment planning is critical to ensuring that decisions around take-home doses accurately reflect their impact on people in opioid agonist treatment.

### Supplementary Information

**Additional file 1.** Completed reporting checklists.

**Additional file 2.** Sample search strategy.

## Data Availability

All data generated or analyzed during this study are included in this published article, its supplementary information files, or the OSF data repository [55].
