# Supplementary material for: The impact of relaxing restrictions on take-home doses during the COVID-19 pandemic on program effectiveness and client experiences in opioid agonist treatment: a mixed methods systematic review
Source: Subst Abuse Treat Prev Policy. 2023 Sep 30;18:56. doi: 10.1186/s13011-023-00564-9 (PMC10543348; doi:10.1186/s13011-023-00564-9)
Supplement: Supplementary file 1 — Additional file 1. Completed reporting checklists. [file 13011_2023_564_MOESM1_ESM.docx]

Additional File 1: Completed reporting checklists

Supplementary Table 1­. Completed PRISMA 2020 checklist . . . p. 2

Supplementary Table 2­. Completed PRISMA-A checklist (PRISMA extension for

abstracts) . . . p. 5

Supplementary Table 3. Completed PRISMA-S checklist (PRISMA extension for reporting literature searches in systematic reviews) . . . p. 6

Supplementary Table 4. Completed SWiM (Synthesis Without Meta-analysis) checklist . . . p. 8

Supplementary Table 1. Completed PRISMA 2020 checklist

| **Topic** | **No.** | **Item** | **Location where item is reported** |
| --- | --- | --- | --- |
| **TITLE** |  |  |  |
| **Title** | 1 | Identify the report as a systematic review. | Title |
| **ABSTRACT** |  |  |  |
| **Abstract** | 2 | See the PRISMA 2020 for Abstracts checklist |  |
| **INTRODUCTION** |  |  |  |
| **Rationale** | 3 | Describe the rationale for the review in the context of existing knowledge. | Introduction |
| **Objectives** | 4 | Provide an explicit statement of the objective(s) or question(s) the review addresses. | Methods |
| **METHODS** |  |  |  |
| **Eligibility criteria** | 5 | Specify the inclusion and exclusion criteria for the review and how studies were grouped for the syntheses. | Table 2 |
| **Information sources** | 6 | Specify all databases, registers, websites, organisations, reference lists and other sources searched or consulted to identify studies. Specify the date when each source was last searched or consulted. | Methods – Search strategy |
| **Search strategy** | 7 | Present the full search strategies for all databases, registers and websites, including any filters and limits used. | OSF repository – Search strategies |
| **Selection process** | 8 | Specify the methods used to decide whether a study met the inclusion criteria of the review, including how many reviewers screened each record and each report retrieved, whether they worked independently, and if applicable, details of automation tools used in the process. | Methods - Screening, data extraction, and critical appraisal |
| **Data collection process** | 9 | Specify the methods used to collect data from reports, including how many reviewers collected data from each report, whether they worked independently, any processes for obtaining or confirming data from study investigators, and if applicable, details of automation tools used in the process. | Methods - Screening, data extraction, and critical appraisal |
| **Data items** | 10a | List and define all outcomes for which data were sought. Specify whether all results that were compatible with each outcome domain in each study were sought (e.g. for all measures, time points, analyses), and if not, the methods used to decide which results to collect. | Methods – Design; Methods – Quantitative synthesis |
|  | 10b | List and define all other variables for which data were sought (e.g. participant and intervention characteristics, funding sources). Describe any assumptions made about any missing or unclear information. | OSF repository - Data extraction form |
| **Study risk of bias assessment** | 11 | Specify the methods used to assess risk of bias in the included studies, including details of the tool(s) used, how many reviewers assessed each study and whether they worked independently, and if applicable, details of automation tools used in the process. | Methods - Screening, data extraction, and critical appraisal |
| **Effect measures** | 12 | Specify for each outcome the effect measure(s) (e.g. risk ratio, mean difference) used in the synthesis or presentation of results. | NA; direction of effect used as standardized metric of effect (see Methods – Quantitative synthesis for justification) |
| **Synthesis methods** | 13a | Describe the processes used to decide which studies were eligible for each synthesis (e.g. tabulating the study intervention characteristics and comparing against the planned groups for each synthesis (item 5)). | Methods - Screening, data extraction, and critical appraisal |
|  | 13b | Describe any methods required to prepare the data for presentation or synthesis, such as handling of missing summary statistics, or data conversions. | Methods – Quantitative synthesis |
|  | 13c | Describe any methods used to tabulate or visually display results of individual studies and syntheses. | Methods – Quantitative synthesis |
|  | 13d | Describe any methods used to synthesize results and provide a rationale for the choice(s). If meta-analysis was performed, describe the model(s), method(s) to identify the presence and extent of statistical heterogeneity, and software package(s) used. | Methods – Quantitative synthesis |
|  | 13e | Describe any methods used to explore possible causes of heterogeneity among study results (e.g. subgroup analysis, meta-regression). | Methods – Quantitative synthesis |
|  | 13f | Describe any sensitivity analyses conducted to assess robustness of the synthesized results. | Methods – Quantitative synthesis |
| **Reporting bias assessment** | 14 | Describe any methods used to assess risk of bias due to missing results in a synthesis (arising from reporting biases). | Methods - Screening, data extraction, and critical appraisal |
| **Certainty assessment** | 15 | Describe any methods used to assess certainty (or confidence) in the body of evidence for an outcome. | NA (did not appraise certainty of evidence; see ‘Methods – Certainty of Evidence’ for justification) |
| **RESULTS** |  |  |  |
| **Study selection** | 16a | Describe the results of the search and selection process, from the number of records identified in the search to the number of studies included in the review, ideally using a flow diagram. | Figure 1 |
|  | 16b | Cite studies that might appear to meet the inclusion criteria, but which were excluded, and explain why they were excluded. | OSF repository – Examples of excluded studies |
| **Study characteristics** | 17 | Cite each included study and present its characteristics. | Tables 3­–4 |
| **Risk of bias in studies** | 18 | Present assessments of risk of bias for each included study. | Tables 6,8,10,12,14, 16­–19. |
| **Results of individual studies** | 19 | For all outcomes, present, for each study: (a) summary statistics for each group (where appropriate) and (b) an effect estimate and its precision (e.g. confidence/credible interval), ideally using structured tables or plots. | Tables 5,7,9,11,13. |
| **Results of syntheses** | 20a | For each synthesis, briefly summarise the characteristics and risk of bias among contributing studies. | Tables 6,8,10,12,14, 16­–19. |
|  | 20b | Present results of all statistical syntheses conducted. If meta-analysis was done, present for each the summary estimate and its precision (e.g. confidence/credible interval) and measures of statistical heterogeneity. If comparing groups, describe the direction of the effect. | Results – Quantitative synthesis; Figures 3­­­–5 |
|  | 20c | Present results of all investigations of possible causes of heterogeneity among study results. | Figures 4­–5 |
|  | 20d | Present results of all sensitivity analyses conducted to assess the robustness of the synthesized results. | Results – Quantitative synthesis – Subgroup analysis - Figure 5 |
| **Reporting biases** | 21 | Present assessments of risk of bias due to missing results (arising from reporting biases) for each synthesis assessed. | Tables 6,8,10,12,14, 16­–19 (MMAT Q3). |
| **Certainty of evidence** | 22 | Present assessments of certainty (or confidence) in the body of evidence for each outcome assessed. | NA (did not appraise certainty of evidence; see ‘Methods – Certainty of Evidence’ for justification) |
| **DISCUSSION** |  |  |  |
| **Discussion** | 23a | Provide a general interpretation of the results in the context of other evidence. | Discussion |
|  | 23b | Discuss any limitations of the evidence included in the review. | Discussion |
|  | 23c | Discuss any limitations of the review processes used. | Discussion |
|  | 23d | Discuss implications of the results for practice, policy, and future research. | Discussion, Conclusions |
| **OTHER INFORMATION** |  |  |  |
| **Registration and protocol** | 24a | Provide registration information for the review, including register name and registration number, or state that the review was not registered. | Methods - Design |
|  | 24b | Indicate where the review protocol can be accessed, or state that a protocol was not prepared. | Methods - Design |
|  | 24c | Describe and explain any amendments to information provided at registration or in the protocol. | Discussion – Deviations from protocol |
| **Support** | 25 | Describe sources of financial or non-financial support for the review, and the role of the funders or sponsors in the review. | Acknowledgements |
| **Competing interests** | 26 | Declare any competing interests of review authors. | Declaration of competing interests |
| **Availability of data, code and other materials** | 27 | Report which of the following are publicly available and where they can be found: template data collection forms; data extracted from included studies; data used for all analyses; analytic code; any other materials used in the review. | Data availability statement |

*From:* Page MJ, McKenzie JE, Bossuyt PM, Boutron I, Hoffmann TC, Mulrow CD, et al. The PRISMA 2020 statement: an updated guideline for reporting systematic reviews. MetaArXiv. 2020, September 14. DOI: 10.31222/osf.io/v7gm2. For more information, visit: [www.prisma-statement.org](https://word-edit.officeapps.live.com/we/www.prisma-statement.org)

Supplementary Table 2. Completed PRISMA-A checklist (PRISMA extension for abstracts).

| **Topic** | **No.** | **Item** | **Reported?** |
| --- | --- | --- | --- |
| **TITLE** |  |  |  |
| **Title** | 1 | Identify the report as a systematic review. | Yes |
| **BACKGROUND** |  |  |  |
| **Objectives** | 2 | Provide an explicit statement of the main objective(s) or question(s) the review addresses. | Yes |
| **METHODS** |  |  |  |
| **Eligibility criteria** | 3 | Specify the inclusion and exclusion criteria for the review. | Inclusion criteria: yes; Exclusion criteria: no (space limitations; refer to Table 2). |
| **Information sources** | 4 | Specify the information sources (e.g. databases, registers) used to identify studies and the date when each was last searched. | Yes (date range) |
| **Risk of bias** | 5 | Specify the methods used to assess risk of bias in the included studies. | Yes |
| **Synthesis of results** | 6 | Specify the methods used to present and synthesize results. | Yes |
| **RESULTS** |  |  |  |
| **Included studies** | 7 | Give the total number of included studies and participants and summarise relevant characteristics of studies. | Total number of participants cannot be calculated (not reported in all primary studies) |
| **Synthesis of results** | 8 | Present results for main outcomes, preferably indicating the number of included studies and participants for each. If meta-analysis was done, report the summary estimate and confidence/credible interval. If comparing groups, indicate the direction of the effect (i.e. which group is favoured). | Yes (to the extent possible in the space permitted) |
| **DISCUSSION** |  |  |  |
| **Limitations of evidence** | 9 | Provide a brief summary of the limitations of the evidence included in the review (e.g. study risk of bias, inconsistency and imprecision). | Yes |
| **Interpretation** | 10 | Provide a general interpretation of the results and important implications. | Yes |
| **OTHER** |  |  |  |
| **Funding** | 11 | Specify the primary source of funding for the review. | No (not possible given word count; refer to funding statement provided in manuscript) |
| **Registration** | 12 | Provide the register name and registration number. | Yes |

Supplementary Table 3. Completed PRISMA-S checklist (PRISMA extension for reporting literature searches in systematic reviews).

| **Section/topic** | **#** | **Checklist item** | **Location(s) Reported** |
| --- | --- | --- | --- |
| **INFORMATION SOURCES AND METHODS** | | | |
| Database name | 1 | Name each individual database searched, stating the platform for each. | Methods – Search strategy |
| Multi-database searching | 2 | If databases were searched simultaneously on a single platform, state the name of the platform, listing all of the databases searched. | n.a. |
| Study registries | 3 | List any study registries searched. | Methods – Search strategy |
| Online resources and browsing | 4 | Describe any online or print source purposefully searched or browsed (e.g., tables of contents, print conference proceedings, web sites), and how this was done. | Methods – Search strategy; OSF repository – Search strategies |
| Citation searching | 5 | Indicate whether cited references or citing references were examined, and describe any methods used for locating cited/citing references (e.g., browsing reference lists, using a citation index, setting up email alerts for references citing included studies). | Methods – Search strategy |
| Contacts | 6 | Indicate whether additional studies or data were sought by contacting authors, experts, manufacturers, or others. | Discussion – Deviations from protocol |
| Other methods | 7 | Describe any additional information sources or search methods used. | Methods – Search strategy |
| **SEARCH STRATEGIES** | | | |
| Full search strategies | 8 | Include the search strategies for each database and information source, copied and pasted exactly as run. | OSF repository – Search strategies |
| Limits and restrictions | 9 | Specify that no limits were used, or describe any limits or restrictions applied to a search (e.g., date or time period, language, study design) and provide justification for their use. | Methods – Search strategy |
| Search filters | 10 | Indicate whether published search filters were used (as originally designed or modified), and if so, cite the filter(s) used. | OSF repository – Search strategies |
| Prior work | 11 | Indicate when search strategies from other literature reviews were adapted or reused for a substantive part or all of the search, citing the previous review(s). | Methods – Search strategy |
| Updates | 12 | Report the methods used to update the search(es) (e.g., rerunning searches, email alerts). | Methods – Search strategy |
| Dates of searches | 13 | For each search strategy, provide the date when the last search occurred. | OSF repository – Search strategies |
| **PEER REVIEW** | | | |
| Peer review | 14 | Describe any search peer review process. | Methods – Search strategy |
| **MANAGING RECORDS** | | | |
| Total Records | 15 | Document the total number of records identified from each database and other information sources. |  |
| Deduplication | 16 | Describe the processes and any software used to deduplicate records from multiple database searches and other information sources. | Methods, Fig. 1 (PRISMA flow chart) |
|  |  |  |  |

Rethlefsen ML, Kirtley S, Waffenschmidt S, Ayala AP, Moher D, Page MJ, Koffel JB, PRISMA-S Group.

Last updated February 27, 2020.

Supplementary Table 4. Completed SWiM (Synthesis Without Meta-analysis) checklist

| **SWiM reporting item** | **Item description** | **Section of manuscript where item is reported*** | **Other**** |
| --- | --- | --- | --- |
| **METHODS** |  |  |  |
| **1 Grouping studies for synthesis** | 1a) Provide a description of, and rationale for, the groups used in the synthesis (e.g., groupings of populations, interventions, outcomes, study design) | Methods - Quantitative synthesis | n.a. |
|  | 1b) Detail and provide rationale for any changes made subsequent to the protocol in the groups used in the synthesis | n.a. | n.a. |
| 2 Describe the standardised metric and transformation methods used | Describe the standardised metric for each outcome. Explain why the metric(s) was chosen, and describe any methods used to transform the intervention effects, as reported in the study, to the standardised metric, citing any methodological guidance consulted | Methods - Quantitative synthesis | n.a. |
| 3 Describe the synthesis methods | Describe and justify the methods used to synthesise the effects for each outcome when it was not possible to undertake a meta-analysis of effect estimates | Methods - Quantitative synthesis | n.a. |
| 4 Criteria used to prioritise results for summary and synthesis | Where applicable, provide the criteria used, with supporting justification, to select the particular studies, or a particular study, for the main synthesis or to draw conclusions from the synthesis (e.g., based on study design, risk of bias assessments, directness in relation to the review question) | n.a. | n.a. |
| 5 Investigation of heterogeneity in reported effects | State the method(s) used to examine heterogeneity in reported effects when it was not possible to undertake a meta-analysis of effect estimates and its extensions to investigate heterogeneity | Methods - Quantitative synthesis | n.a. |
| 6 Certainty of evidence | Describe the methods used to assess certainty of the synthesis findings | NA (did not appraise certainty of evidence; see ‘Methods – Certainty of Evidence’ for justification) | n.a. |
| 7 Data presentation methods | Describe the graphical and tabular methods used to present the effects (e.g., tables, forest plots, harvest plots). Specify key study characteristics (e.g., study design, risk of bias) used to order the studies, in the text and any tables or graphs, clearly referencing the studies included | Methods - Quantitative synthesis | n.a. |
| **RESULTS** |  |  |  |
| 8 Reporting results | For each comparison and outcome, provide a description of the synthesised findings, and the certainty of the findings. Describe the result in language that is consistent with the question the synthesis addresses, and indicate which studies contribute to the synthesis | Results – Quantitative synthesis | n.a. |
| **DISCUSSION** |  |  |  |
| 9 Limitations of the synthesis | Report the limitations of the synthesis methods used and/or the groupings used in the synthesis, and how these affect the conclusions that can be drawn in relation to the original review question | Discussion | n.a. |

The citation for the Synthesis Without Meta-analysis explanation and elaboration article is: Campbell M, McKenzie JE, Sowden A, Katikireddi SV, Brennan SE, Ellis S, Hartmann-Boyce J, Ryan R, Shepperd S, Thomas J, Welch V, Thomson H. Synthesis without meta-analysis (SWiM) in systematic reviews: reporting guideline BMJ 2020;368:l6890 http://dx.doi.org/10.1136/bmj.l6890

*The original SWiM guideline calls for the page number to be reported. We have specified ‘section of the manuscript’ because page numbers may not be present when accessing material electronically.
**If the information is not provided in the systematic review, give details of where this information is available (e.g., protocol, other published papers (provide citation details), or website (provide the URL)).
