# Supplementary material for: The impact of relaxing restrictions on take-home doses during the COVID-19 pandemic on program effectiveness and client experiences in opioid agonist treatment: a mixed methods systematic review
Source: Subst Abuse Treat Prev Policy. 2023 Sep 30;18:56. doi: 10.1186/s13011-023-00564-9 (PMC10543348; doi:10.1186/s13011-023-00564-9)
Supplement: Supplementary file 2 — Additional file 2. Sample search strategy. [file 13011_2023_564_MOESM2_ESM.docx]

Additional File 2: Sample search strategy

**DATABASE**: Medline(R)ALL (Ovid)

**SEARCH STRATEGY:** (Set #1a OR Set #1b) AND (Set #2a OR Set #2b) AND Publication Year = 2020-current

**Set #1: Population (people receiving opioid agonist treatment)**

*a. OAT and OAT medications*

Opiate substitution treatment/ OR Opioid-Related Disorders/dt OR Heroin Dependence/dt OR (opioid substitution OR opiate substitution OR heroin substitution OR opioid replacement OR opiate replacement OR heroin replacement OR opioid maintenance OR opiate maintenance OR heroin maintenance OR opioid assisted treatment* OR opiate assisted treatment* OR heroin assisted treatment* OR medication assisted treatment* OR drug assisted treatment* OR opioid agonist* OR opiate agonist* OR agonist opioid treatment* OR substitution treatment*).ti,ab,kf

OR exp Buprenorphine/ OR Methadone/ OR Heroin/tu OR (buprenorphine OR Suboxone OR methadone OR Methadose OR Metadol-D OR Dolophine OR ((heroin OR diamorphine OR diacetylmorphine) AND (prescription* OR prescrib*))).ti,ab,kf

*b. Treatment for opioid use disorders*

(Opioid-Related Disorders/ OR Heroin Dependence/ OR Morphine Dependence/ OR Opium Dependence/ OR ("substance use disorder" OR "substance use disorders" OR substance user* OR substance abuse* OR substance misuse* OR substance dependen* OR substance disorder* OR "drug use disorder" OR "drug use disorders" OR drug user* OR drug abuse* OR drug misuse* OR drug dependen* OR "opioid use disorder" OR "opioid use disorders" OR opioid user* OR opioid abuse* OR opioid misuse* OR opioid dependen* OR opiate user* OR opiate dependen* OR "opiate use disorder" OR "opiate use disorders" OR "heroin use" OR heroin user* OR heroin abuse* OR heroin misuse* OR heroin dependen* OR "narcotic use" OR narcotic user* OR narcotic abuse* OR narcotic misuse* OR narcotic dependen* OR addict* OR "people who inject drugs" OR PWID OR "people who use drugs" OR PWUD or "people with lived experience" OR "people with lived and living experience" OR PWLLE).ti,ab,kf)

AND ((treatment* OR maintenance therap* OR replacement therap* OR medication* OR substitution OR drug replacement OR OAT OR iOAT OR tiOAT OR MMT OR HAT OR ORT OR OST OR DAM OR MAT OR MOUD).ti,ab,kf)

**Set #2: Intervention/Context (relaxation of restrictions on take-home doses during Covid-19 pandemic)**

*a. Take-home doses*

(carries or carry-home* or take-home* or home dos* or takeaway* or take-away* or supervised or unsupervised or witnessed or unwitnessed or unobserved or observed dos*).ti,ab,kf

*b. Covid-19 pandemic*

Covid-19/ OR SARS-CoV-2/ OR (covid* OR SARS-CoV-2 OR SARS-COV2 OR coronavirus* OR corona virus* OR pandemic* OR global outbreak*).ti,ab,kf
